# An electronic nose can identify humans by the smell of their ear

**DOI:** 10.1093/chemse/bjad053

**Published:** 2024-01-18

**Authors:** Stephanie Brener, Kobi Snitz, Noam Sobel

**Affiliations:** The Azrieli National Center for Human Brain Imaging and Research, Weizmann Institute of Science, Rehovot 7610001, Israel; The Department for Brain Sciences, Weizmann Institute of Science, Rehovot 7610001, Israel; The Azrieli National Center for Human Brain Imaging and Research, Weizmann Institute of Science, Rehovot 7610001, Israel; The Department for Brain Sciences, Weizmann Institute of Science, Rehovot 7610001, Israel; The Azrieli National Center for Human Brain Imaging and Research, Weizmann Institute of Science, Rehovot 7610001, Israel; The Department for Brain Sciences, Weizmann Institute of Science, Rehovot 7610001, Israel

**Keywords:** electronic nose, body odor, smell, cerumen, ear, biometrics

## Abstract

Terrestrial mammals identify conspecifics by body odor. Dogs can also identify humans by body odor, and in some instances, humans can identify other humans by body odor as well. Despite the potential for a powerful biometric tool, smell has not been systematically used for this purpose. A question arising in the application of smell to biometrics is which bodily odor source should we measure. Breath is an obvious candidate, but the associated humidity can challenge many sensing devices. The armpit is also a candidate source, but it is often doused in cosmetics. Here, we test the hypothesis that the ear may provide an effective source for odor-based biometrics. The inside of the ear has relatively constant humidity, cosmetics are not typically applied inside the ear, and critically, ears contain cerumen, a potent source of volatiles. We used an electronic nose to identify 12 individuals within and across days, using samples from the armpit, lower back, and ear. In an identification setting where chance was 8.33% (1 of 12), we found that we could identify a person by the smell of their ear within a day at up to ~87% accuracy (~10 of 12, binomial *P* < 10^−5^), and across days at up to ~22% accuracy (~3 of 12, binomial *P* < 0.012). We conclude that humans can indeed be identified from the smell of their ear, but the results did not imply a consistent advantage over other bodily odor sources.

## Introduction

Biometric identification has grown to encompass a diverse repertoire of methods. Older methods such as fingerprinting and retinal scanning have been augmented with newer tools such as facial, gate, and voice recognition. Whereas visual and auditory source information is applied extensively in biometrics (Andrijchuk et al. 2005; Takayuki 2019), olfactory information is not. This technological lack is in sharp contrast to animal behavior. Terrestrial mammals can effectively identify conspecifics by body odor (Brennan and Kendrick 2006), suggesting that human body odor may provide an added measure for biometrics. Humans, in turn, exhibit mostly rudimentary capabilities in identifying conspecifics by smell. They can identify themselves at just above chance levels (Russell 1976; Hold and Schleidt 1977; Olsson et al. 2006), with women outperforming men (Schleidt et al. 1981). Humans can also identify their own offspring in multiple-alternative forced-choice tests (Porter and Moore 1981; Kaitz et al. 1987; Weisfeld et al. 2003), and can identify more distant kin in 2 alternative forced-choice tests (Porter et al. 1986). However, only about a third of humans can identify their regular sexual partners by body odor (Hold and Schleidt 1977; Schleidt et al. 1981). Curiously, this ability to identify partners by body odor is 2-fold better in women who experience repeated spontaneous unexplained pregnancy loss, a pattern that echoes the rodent Bruce effect (Rozenkrantz et al. 2020). Finally, humans can also identify their non-romantic friends by body odor (Olsson et al. 2006), and, in fact, may initiate friendships in part based on similarity in body odor (Ravreby et al. 2022). In the majority of the above instances, however, testing was in the form of two- or multiple-alternative forced-choice tests rather than biometric-type identification out of a large pool of candidates. Does this rudimentary performance imply that biometric-quality information is unavailable in human body odor, or rather that humans are simply not fully tuned to this information? Several lines of evidence point to the latter. First, in contrast to humans, dogs are in fact highly capable of identifying humans by smell (Hepper 1988; Pinc et al. 2011; Marchal et al. 2016; Woidtke et al. 2018; Filetti et al. 2019), even when the odor was collected from across body regions (Schoon and De Bruin 1994). Further evidence for individual olfactory fingerprints is available from gas-chromatography mass-spectrometry (GCMS) studies of human body odor (Bernier et al. 1999; Haze et al. 2001; Curran et al. 2005a, 2005b, 2007). A particularly large study on axillary sweat, saliva, and urine samples from 197 adults found that members of the same family had more similar GCMS fingerprints to one another than to members of other families. Moreover, they found significant similarity in GCMS fingerprints per individual when comparing repeat samples of the same individual versus other participants (Penn et al. 2007). In a separate study, this approach enabled individual identification of 10 humans by the smell of their hand alone using GCMS (Curran et al. 2010). GCMS may also provide information beyond identification alone, such as body odor fingerprints associated with differing emotional states (Smeets et al. 2020).

Whereas GCMS may allow for human olfactory fingerprinting, it is not a practical tool, primarily due to its cost, size, and complexity of operation. A practical alternative is in the sensing platform typically referred to as an electronic nose (eNose), namely an array of chemical sensors with various sensitivities that generate odorant-specific patterns (Persaud and Dodd 1982; Hodgins 1995; Schaller et al. 1998; James et al. 2005; Röck et al. 2008; Wilson and Baietto 2009; Karakaya et al. 2020; Khatib and Haick et al. 2022). Although there are many flavors of eNose, we know of only 3 pilot efforts to identify humans using these devices. An initial effort used cotton swabs to sample body odor from the armpits of 2 participants and later measured these samples offline with a lab-constructed eNose containing 5 different metal oxide sensors (Wongchoosuk et al. 2009). The authors reported successful discrimination between the 2 participants in a PCA plot. In an ensuing effort by the same group, the authors switched to online rather than offline sampling and reported successful discrimination between 4 individuals from armpit odor with 95% confidence (Wongchoosuk et al. 2011). Finally, a third effort entailed a wearable eNose containing 6 functionalized carbon nanotube sensors measuring from the armpit, and it was worn by 8 participants for 63 min each (Zheng et al. 2019). Using all the data, the authors reached 91.67% identification accuracy. Notably, all these previous efforts did not tackle new data obtained on a day different from the training data. In sum, the overall data on human identification by eNose is very limited.

Two primary questions when setting out to identify humans by eNose are which eNose to use, and which bodily odor source to sample. This study relates to the latter. An obvious candidate for sampling is breath. Given that breath is a transfer media between the inside of the body and the outside world and is subject to metabolic bodily processes, it is potentially an ideal candidate. Indeed, breath is the primarily targeted media in various eNose-based disease detection efforts (Dragonieri et al. 2007, 2017; Fens et al. 2009; De Vries et al. 2015; Scarlata et al. 2015; Wilson, 2015; Nardi Agmon et al. 2022). However, breath is also dramatically impacted by transient events, such as diet, and contains very high and variable humidity. Humidity is the primary enemy of many eNose platforms (James et al. 2005; Kashwan and Bhuyan, 2005; Röck et al. 2008) because water adsorbed on the sensing surface increases the resistance of the sensing layers and blocks the reaction site, causing gas sensor response drift (Abdullah et al. 2022). A second obvious candidate is armpit, which was indeed the target of the above-noted eNose efforts. The armpit, however, is often doused in cosmetics. Although such cosmetics may themselves provide relevant information (Allen and Roberts 2015), they may nevertheless stand in the way of individual fingerprinting. By contrast, in this study, we propose an alternative: the inside of the ear. The ear is easily accessible, it is not particularly humid (nor does its humidity fluctuate with the respiratory cycle), and the inside of the ear is not typically subjected to cosmetics (in contrast to the outside back of the ear). Most critically, the inside ear contains cerumen, a known source of body volatiles (Preti and Leyden 2010; Harker et al. 2014; Shokry et al. 2017). Moreover, an initial GCMS study identified 12 cerumen volatiles that can discriminate between East Asians and non-Asians (Prokop-Prigge et al. 2014), and a second GCMS study used cerumen to discriminate between African, Caucasian, and Asian descent participants (Prokop-Prigge et al. 2015). These ethnic specificities likely reflect differing overall quantities/intensity of cerumen, a pattern that has been linked to the ABCC11 gene (Preti and Leyden 2010; Harker et al. 2014). This, combined with the existence of volatile disease markers in cerumen (Barbosa et al. 2019), together suggests that this media may be a promising candidate for individual identification by smell.

Here, we will address this hypothesis using an AirSense PEN3 eNose. This commercial device consists of a gas sampling unit and a sensor array which is composed of 10 thermo-regulated metal oxide sensors. Each sensor has a unique coating, making it sensitive to a particular set of chemical compounds. When a compound interacts with the sensor, the resulting oxygen exchange causes decreased electrical conductivity. These changes are seen in a 10-channel time series from which we can extract meaningful information about the odor. We used this device to sample 12 individuals once a day for 5 days. Each sampling session contained 3 samples from each of 3 body regions: the armpit, ear, and lower back. We asked 2 questions: can we identify participants from the odor of their ear, and does ear outperform armpit and lower back.

## Methods

### Participants

Twelve healthy adults were selected for this study with no other exclusion criteria (8F, 4M, mean age 30.5 ± 7.8 years). The 12 individuals came to the lab for 5 consecutive days (Sunday to Thursday) where their ear, armpit, and lower back were sampled by 2 eNoses 3 times each on each day. All participants provided informed written consent to procedures approved by the Weizmann Institute of Science Institutional Review Board, in compliance with the declaration of Helsinky for Medical Research involving human subjects. Participants were paid 50 NIS (~14 Euro) per day, leading to a total of 250 NIS (~70 Euro) paid in full on the last day.

### eNose setup

We used 2 PEN3 eNoses (AIRSENSE Analytics GmbH, Schwerin, Germany). The PEN3 consists of a gas sampling unit and a sensor array. The sensor array is made of 10 different thermo-regulated metal oxide sensors held in a stainless-steel chamber (volume: 1.8 mL, temperature: 110°C). Each sensor is coated in a unique material that makes it sensitive to different sets of chemical compounds. The sensitivities of each sensor can be seen in Table 1 (From the manufacturer, recreated in Zheng and Wang 2006; Wu et al. 2017). When a compound interacts with the surface of the sensor, the oxygen exchange that occurs causes a change in electrical conductivity. This conductivity is the unit of measurement displayed in the time series produced by the device. We used the PEN3 with its native software (Winmuster).

**Table 1.** Sensors in the PEN3 eNose and their sensitivities. Table adapted from Wu et al. (2017) and Zheng and Wang (2006) (sourced from AirSense manufacturer data).

Number in array	Sensor name	General description	Reference, mL m^−3^ (ppm)
1	W1C	Aromatic organic compounds	Toluene, 10
2	W5S	Very sensitive, broad range sensitivity, reacts to nitrogen oxides, very sensitive with a negative signal	NO_2_, 1
3	W3C	Ammonia, also used as a sensor for aromatic compounds	Benzene, 10
4	W6S	Detects mainly hydrogen gas	H2, 0.1
5	W5C	Alkanes, aromatic compounds, and nonpolar organic compounds	Propane, 1
6	W1S	Sensitive to methane. A broad range of organic compounds detected	CH_3_, 100
7	W1W	Detects inorganic sulfur compounds, for example, H_2_S. Also sensitive to many terpenes and sulfur-containing organic compounds	H_2_S, 1
8	W2S	Detects alcohol, partially sensitive to aromatic compounds, broad range	CO, 100
9	W2W	Aromatic compounds, inorganic sulfur, and organic compounds	H_2_S, 1
10	W3S	Reacts to high concentrations (>100 mg kg^−1^) of methane and aliphatic organic compounds	CH_3_, 100

Two eNoses were used simultaneously in this experiment. Both eNoses used the following parameters: Measurement time = 50 s, Flush time = 40 s, Zero-point trim time = 10 s. eNose 1 was given a Chamber flow and Initial injection flow of 400 mL/min, and eNose 2 was given a Chamber flow and Initial injection flow of 600 mL/min. Both eNoses were connected via USB to 2 separate computers. To run a measurement on both eNoses, the experimenter pressed “play” on both Winmuster interfaces simultaneously. This would start an entire eNose cycle, which for this experiment consisted of a 40 s Flush phase, 10 s Baseline phase, and a 50 s Measurement phase, for a total of 100 s per measurement. Each measurement from each eNose was then named and saved to its respective computer.

### Sampling cup and tip

**Fig. 1.**
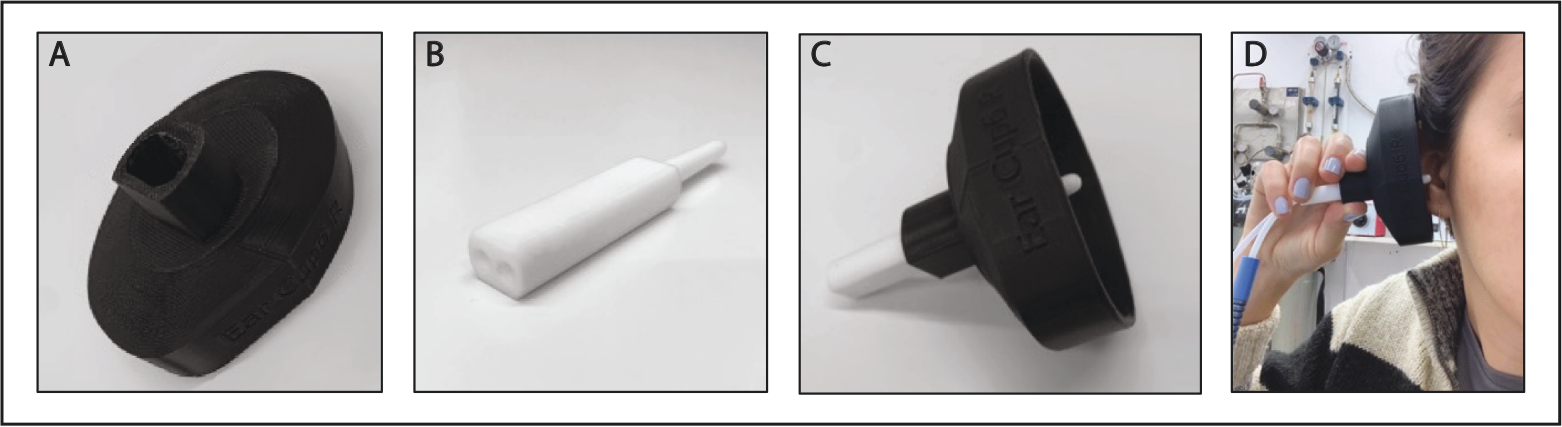
The sampling cup and tip. A) The sampling cup alone. B) The sampling tip alone. C) The cup and tip combined. D) The apparatus applied to the ear with both eNose hoses attached.

We developed a device that combined both eNose sampling tubes into one Teflon probe that was placed at the opening of the ear canal in a steady and controlled manner (Fig. 1). The device contained a sampling cup that was 3D-printed in a shape that covers the entire ear. This cup allowed for an ear headspace unaffected by room airflow, blocked possible cosmetic VOCs from behind the ear, and held the sampling tip at a fixed location in front of the ear canal. The sampling tip was machined from Teflon to assure minimal odor contamination. The 6 mm diameter tip contained an inner septum such that both eNose flow paths remained independent to the tip. The tip was machined as a perforated ball, with 3 1-mm perforations for each flow path. We prepared an independent cup for each participant and a total of 20 tips for the experiment. In each session, we used a separate tip for each body region, and tips were washed in boiling water and air-pressure dried between uses.

### Control odorant

To later account for potential eNose drift, we collected 2 samples of an identical odorant at the beginning of each session. The 2 samples differed in that one accumulated jar headspace for 12 h before sampling and the other was fresh. This control odorant contained 10 components present in general body odor (de Lacy Costello et al. 2014; Jha et al. 2015; Prokop-Prigge et al. 2016) and in cerumen (Prokop-Prigge et al. 2014). The final dilution contained 1PPM of Hexanoic acid (CAS# 142-62-1), Isobutyric acid (CAS# 79-31-2), Valeric acid (CAS# 109-52-4), Propanoic acid (CAS# 79-09-4), Butyric acid (CAS# 107-92-6), Acetic acid (CAS# 64-19-7), Isovaleric acid (CAS# 503-74-2), 2-methylbutyric acid (CAS# 116-53-0), 6-methyl-5-heptene-2-one (CAS# 110-93-0), and 2 PPM of trans-geranylacetone (CAS# 3796-70-1) in mineral oil. The stock solution was vortexed before use.

### Procedures

**Fig. 2.**
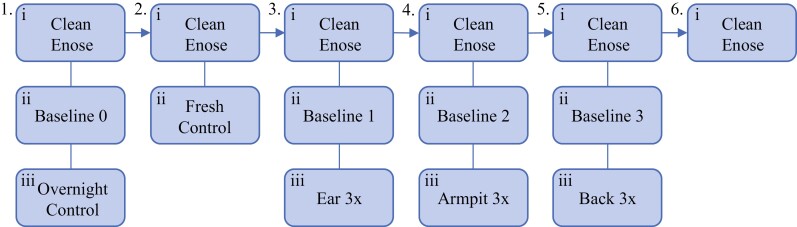
Flow diagram of the experimental protocol for each participant.

Participants were instructed not to wear deodorant or perfume and were provided with identical body soap, shampoo, and conditioner to use throughout the study period such that any cosmetics-associated odors would be constant across the cohort. Each day before the first participant arrived (Supplementary Table 1), the eNoses were turned on and sent through a “clean cycle.” A “clean cycle” refers to running the eNoses through a full 100 s eNose cycle but with the sampling tubes not attached to anything. This is done to clean any residual odor held in the eNose tubes. The eNoses were run on these “clean cycles” as many times as necessary until the measurement showed that all sensors were steady at 1 G0/G. During this time, the fresh control odor was prepared (~5–10 min before sampling it) before each participant. Once clean, the first Baseline 0 was taken. This baseline was done by running the eNoses through another cycle and saving this result. Then the overnight control was sampled followed by the fresh control, using the sampling protocol for eNose vials through a septum cap. The eNose was run through another clean cycle, followed by taking Baseline 1 of the open room air with the sampling cup and tip attached to the eNose tubes. Then the participant’s first body region (either right ear, right armpit, or lower back) (Supplementary Table 2) was sampled 3 times in a row, followed by a clean cycle and taking Baseline 2 with a new tip and the same sampling cup attached. The second body part was measured 3 times with this new tip, followed by another clean cycle and Baseline 3 with another new tip. Finally, the last body part was measured 3 times, and the eNose was run through clean cycles until the next participant came (Fig. 2).

The right ear was sampled by asking the participant to hold the sampling cup and tip up to their ear and ensure the tip did not touch any part of their ear. The right armpit was sampled by adjusting the tip as far out of the cup as possible and helping the participant hold the unit on their armpit. The lower back was sampled with the tip in the same location as the armpit, and then asking the participant to lift their shirt while the experimenter held the unit in place on their back. For each body part, the sampling unit was placed against the body part 5 s before the end of the Baseline Trim phase in the eNose cycle.

### Data analysis

All analyses were conducted using MATLAB software (Mathworks, USA). We used the endpoints of each sensor to condense the 10-channel × 50 s time series for one eNose to a vector of 1 × 10 endpoints. We did this for each of the 2 eNoses, then combined the data from both sets of 10 sensors to create a hybrid dataset of all 20 sensors together yielding 1 × 20 endpoints per sample. Initial analysis was conducted using the previously selected Fine K-nearest neighbors (Fine KNN) classifier (Ravreby et al. 2022). Additional exploratory testing was conducted by entering Day-1 data for each body region into MATLAB’s Classification Learner Toolbox with 36-fold cross-validation (leave-one-out test) where classification was attempted by 24 classifiers. For each body region, we first identified the best classifier. The a-priori selected Fine KNN classifier was indeed best for *ear* data, and the Linear Discriminant model was best for *armpit* and *lower back* data. For each comparison, we then trained and tested the model on both leave-one-out (36-fold cross validation) and 3-fold cross validation (test on 66.7%, train on 33.3%) for 500 iterations. In a leave-one-out validation test, the model was trained on 35 samples and tested on one remaining sample. In a 3-fold cross validation test the model was trained on 2 samples per person and tested on the third sample per each person. After reviewing the initial results, we proceeded to only use the stricter 3-fold cross validation test. We ran the classification 500 times and took the mean value as the true accuracy of the model.

### Accounting for drift

To account for drift, we took regular eNose measurements of the above-described control odorant. An individual control odorant that was made fresh prior to each participant was sampled before measuring each participant. To generate drift-corrected values, all ear, armpit, and lower back data from each participant on any given day was then divided by the matched fresh control odorant endpoint measurements from the corresponding day. This means that all sensor endpoint data of Ear Day 1, Armpit Day 1, and Back Day 1 for Participant 1 were divided by all sensor endpoint data of Fresh Control Odor Day 1 Participant 1, and so on.

### Permuting data and statistical testing

To determine whether the classification accuracy for each body part was statistically significant, we shuffled the class labels, ran the shuffled data through the trained classifiers, and used the resulting accuracy values from 500 iterations to build a null distribution. This was done for both the 3-fold cross validation test as well as for the 36-fold cross validation test. The accuracy values of the shuffled distribution were then compared to the median accuracy from the distribution of real accuracies to obtain the p statistic. The binomial p statistic was then calculated with the following formula: $P(r){\;=\;}^{n}C^{r}\times p^{r}{\left(1-p\right)}^{n-r}$, where the probability (*p*) of a correct outcome by chance is 0.0833, *n* is 12 for the 12 individuals, and the number correct (*r*) is the number out of 12 that yielded the mean accuracy value on the test for which we are computing the statistic.

## Results

We tested real-time sampling of body odors for personal identification using eNose technology. Twelve participants (8F, 4M) had their right *ear*, right *armpit*, and *lower back* sampled 3 times each per session, for 5 consecutive days. Two control odors (fresh and overnight) were sampled before each participant, and a baseline measurement was taken between each body part and control odors. There are 2 primary approaches to treating eNose sensor signals: one is to use the entire time-course, considering its full shape, and the other is to use only the point at which the sensor reaches equilibrium. In the current instance, the latter reduces the 10-sensor 50-s series to a 1 × 10 vector. We explored both approaches, yet to maintain a manageable manuscript extent, we report only on the latter, which produced superior performance in this particular case. Moreover, we simultaneously sampled using 2 technically identical eNoses, each sampling at a slightly different airflow rate (400 mL/min and 600 mL/min). We acknowledge that there are various possible paths to combining these data traces, yet this manuscript is focused not on optimizing eNose methodology, but rather on the question of whether humans can be identified by the smell of their ear, and, therefore, we limit our report to the simple combination of both devices, that is, we treat them as one eNose with 20 sensors, as this approach yielded slightly better results than each eNose alone. We note that all the raw data of this manuscript is available for download in Supplementary Data Set 1, allowing for alternative investigations of the data.

### Individuals can be identified from samples within a day

**Fig. 3.**
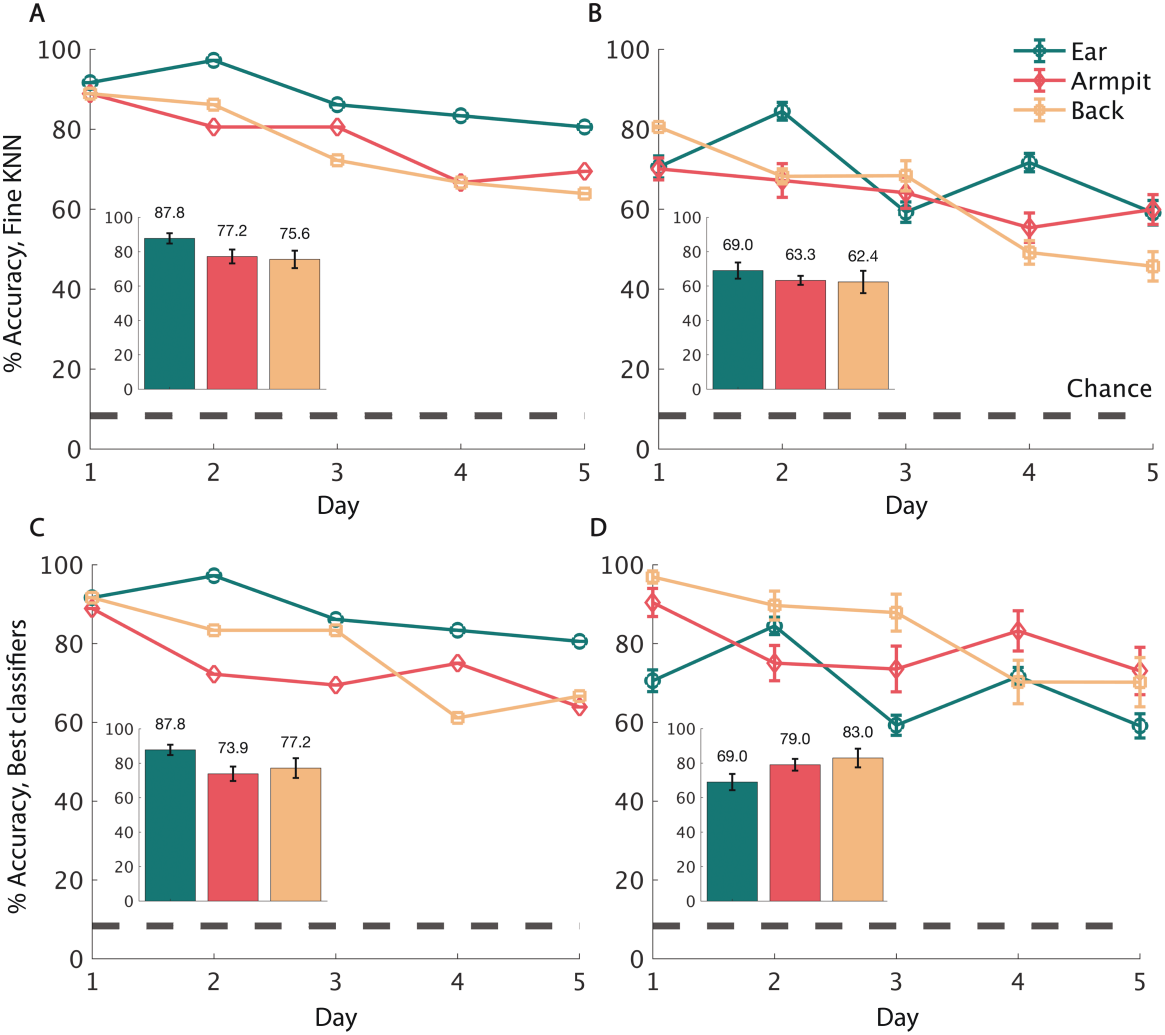
Individuals can be identified across samples within a day. A) Mean classification accuracy of individuals using the drift-corrected odor from ear, armpit, and lower back when trained and tested within single days using the Fine KNN classifier in a leave-one-out test for all body regions. Drift correction is done by dividing each sample by its matched fresh control odor. The overall average accuracy with standard error bars for ear, armpit, and back is shown as an insert in each panel. The chance level prediction accuracy is shown with the dashed black line at 8.3% accuracy. B) Within-day classification accuracy under the same conditions but for the 3-fold cross validation case plotted with standard deviation. C) Mean within-day classification accuracy of individuals using the drift-corrected odor from ear, armpit, and lower back trained with each body region’s best-performing respective classifier in a leave-one-out test for all body regions. D) Within-day classification accuracy under the same conditions but for the 3-fold cross validation test plotted with standard deviation.

We sought to find if, within one day of sampling, the 12 participants could be classified from one another accurately. The data were split into individual days where each day contained three samples per body part per person (36 *ear* samples per day, 36 *armpit* samples per day, 36 *lower back* samples per day). Given drift in eNose signal (see estimate of the drift in Supplementary Fig. 1), we conducted analyses twice: once without and once with correction for drift by calibrating to the prepared control odorant. In a previous study using this same eNose to measure body odor, we found that the KNN classifier provided the strongest outcome (Ravreby et al. 2022), so we, therefore, applied this same classifier here. The chance probability for classifying an individual in this test is 8.33%. We observed that with *k* = 2 neighbors, using a leave-one-out test, the Fine KNN classifier identified individuals from the smell of their *ear* within a single day with an average accuracy of 67.2% without drift-correction (difference from chance, binomial *P* < 10^−5^) (Supplementary Fig. 2A) and 87.8% with correction (difference from chance, binomial *P* < 10^−5^) (Fig. 3A). Using the stricter 3-fold cross validation, identification was 50.9% for uncorrected (difference from chance, binomial *P* < 10^−5^) (Supplementary Fig. 2B) and 69% for drift-corrected data (difference from chance, binomial *P* < 10^−5^) (Fig. 3B). Using the same classifier for *armpit*, with the leave-one-out test, we observe 58.9% for uncorrected (difference from chance, binomial *P* < 10^−5^) and 77.2% for drift-corrected data (difference from chance, binomial *P* < 10^−5^) (Fig. 3A). Using the stricter 3-fold cross validation, identification was 45.5% for uncorrected (difference from chance, binomial *P* < 0.0002) and 63.3% for drift-corrected data (difference from chance, binomial *P* < 10^−5^) (Fig. 3B). Finally, using the same KNN classifier for *lower back* data, with the leave-one-out test, we observe 59.4% for uncorrected (difference from chance, binomial *P* < 10^−5^) and 75.6% for drift-corrected data (difference from chance, binomial *P* < 10^−5^) (Fig. 3A). Using the stricter 3-fold cross validation, identification was 40.7% for uncorrected (difference from chance, binomial *P* < 0.001) and 62.4% for drift-corrected data (difference from chance, binomial *P* < 10^−5^) (Fig. 3B). To directly compare these results, we conducted a one-way analysis of variance (ANOVA) on the 3-fold validated data, with a condition of *body region*. When using the data not corrected for drift, we observe a significant effect (*F* = 770.9, *P* < 10^−5^). Post-hoc tests revealed that *armpit* was better than *lower back* (*P* < 10^−5^, Cohen’s *d* = 1.398), and *ear* was better than both (*ear* vs *armpit*: *P* < 10^−5^, Cohen’s *d* = 1.472, *ear* vs *lower back*: *P* < 10^−5^, Cohen’s *d* = 3.016). When using drift-corrected data, we again observed a significant effect (*F* = 305.6, *P* < 10^−5^). Here, however, post-hoc tests revealed a different order of effectiveness: *lower back* was significantly better than *armpit* (*P* < 10^−5^, Cohen’s *d* = 0.2855), yet *ear* was significantly better than both (*ear* vs *lower back*: *P* < 10^−5^, Cohen’s *d* = 1.784, *ear* vs *armpit*: *P* < 10^−5^, Cohen’s *d* = 2.438). In sum, using the previously applied KNN classifier, within-day classification was better than chance using any body region, both with and without correction for drift. When correcting for drift, the best results were obtained from *ear* data.

Consistent with our hypothesis, the final above analysis using our a-priori selected classifier (Ravreby et al. 2022) implied an advantage for sampling from the *ear*. To gauge the strength of this, we tested whether we could negate this *ear* advantage by identifying an optimal classifier for each body region. We tested the 24 classifiers available in the MATLAB Classification Learner Toolbox. We found that the Linear Discriminant model provided the best results for both *armpit* and *lower back* data. Using a 36-fold cross-validation test on drift-corrected data, the average identification accuracy within 1 day for *armpit* was 73.9% (difference from chance, binomial *P* < 10^−5^), and *lower back* was 77.2% (difference from chance, binomial *P* < 10^−5^) (Fig. 3C). On a 3-fold cross-validation test, the accuracy for *armpit* was 79.1% (difference from chance, binomial *P* < 10^−5^) and *lower back* was 83% (difference from chance, binomial *P* < 10^−5^) (Fig. 3D). We now again conducted a one-way ANOVA with a condition of *body region*, this time using the best classifier for each region. We found a significant effect of *body region* (*F* = 1667, *P* < 10^−5^ on leave one out, and *F* = 1273, *P* < 10^−5^ on 3-fold). Post-hoc tests on the 3-fold condition revealed that *armpit* was better than *ear* (*P*< 10^−5^, Cohen’s *d* = 1.784), and *lower back* was better than both (*lower back* vs *armpit*: *P* < 10^−5^, Cohen’s *d* = 0.8061, *lower back* vs *ear*: *P* < 10^−5^, Cohen’s *d* = 3.722). Using the best classifier we could find for each body region, the *lower back* was now better than the *ear* for classification.

**Fig. 4.**
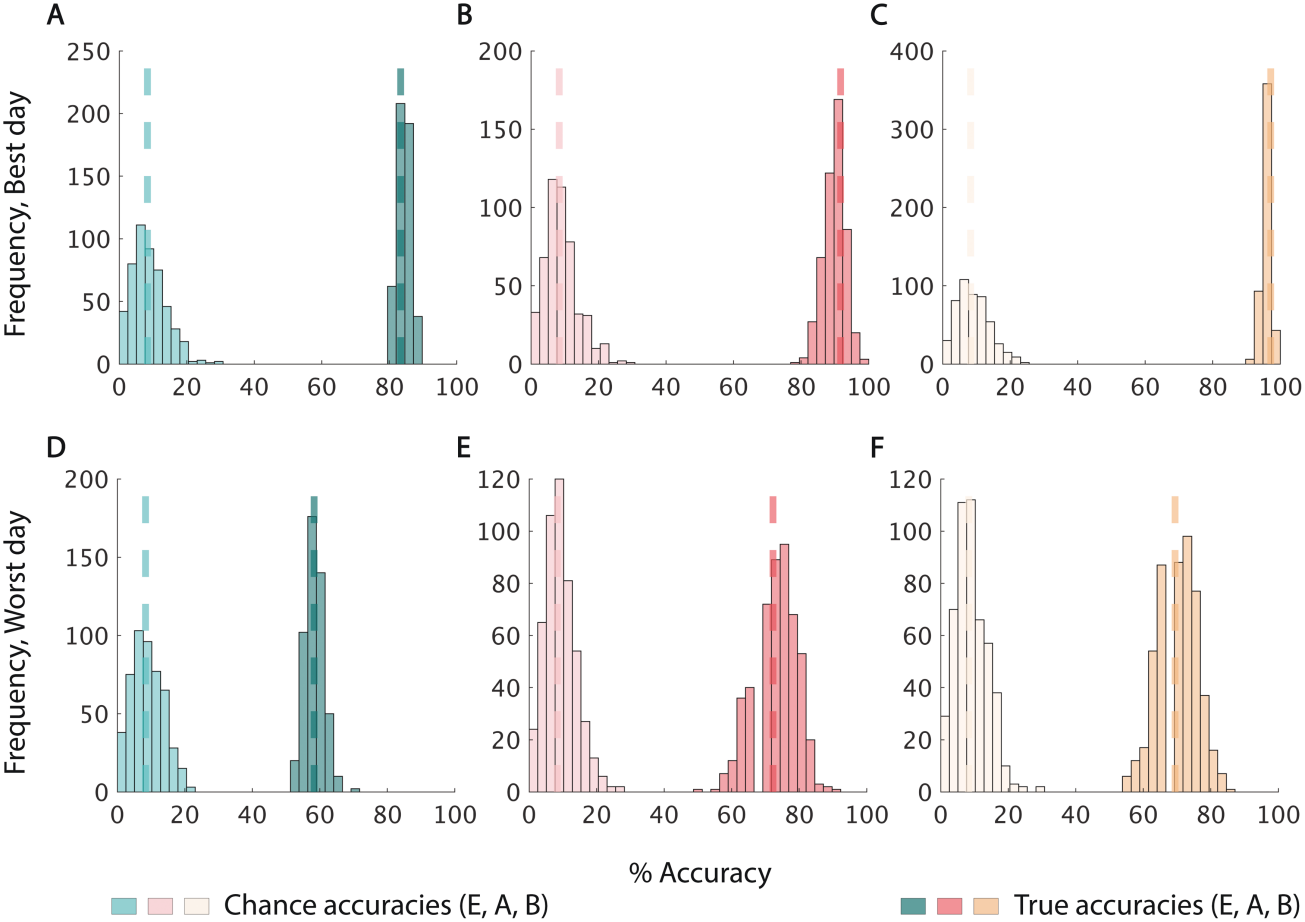
Within single day performance was significantly better than chance. A–C) Distributions of accuracy values on 500 iterations of training/testing with the best respective classifiers on drift-corrected real data on the right and shuffled data on the left for ear, armpit, and lower back, respectively, from the best-performing day. Median accuracy values are displayed in dashed lines. D–F) Distributions of accuracy values under the same conditions but for each body region’s worst-performing day.

With the results of the optimal classifiers in hand, we sought to better evaluate the difference from chance. We compared the median value of 500 iterations of 3-fold classification on real data with the median value of 500 iterations of classification on shuffled data using both the worst-performing day and the best-performing day for each body region respectively. For both uncorrected and drift-corrected data, and on best and worst-performing days for the *ear*, *armpit*, and *lower back*, this test (the Bernoulli probability) yielded a permutation *P*-value = 0.002 (Fig. 4, Supplementary Fig. 3). In conclusion, even on the worst sampling day, the *ear*, *armpit*, and *lower back* all perform significantly better than chance, with or without drift correction.

### Individuals can be identified using samples accumulated over days

**Fig. 5.**
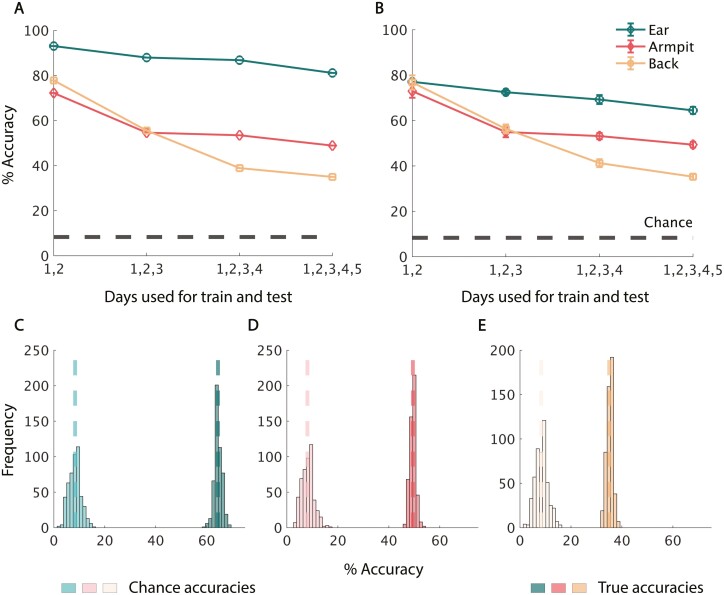
An ear advantage emerges as data is accumulated. A) Mean across accumulated days classification accuracy of individuals using the drift-corrected odor from ear, armpit, and lower back trained and tested on multiple days with each body region’s best-performing respective classifier in a leave-one-out test for all body regions. Standard deviation bars shown. B) Across accumulated days classification accuracy under the same conditions but for the leave-one-sample-per-participant-out cross validation test. C–E) Distributions of accuracy values on 500 iterations of training/testing with a leave-one-value-per-participant-out test on accumulated Days 1, 2, 3, 4, and 5 with the best respective classifiers on real data on the right and shuffled data on the left for ear, armpit, and lower back, respectively. Median accuracy values are displayed in dashed lines.

We next sought to determine whether we can identify people not only within 1 day but using multiple accumulated days. We applied the same analysis with the same optimal classifiers for each region and trained and tested on consecutively aggregated days (e.g. 2 days of data, 3 days, 4 days, and 5 days). Specifically, we conducted both the leave-one-out and the leave-one-sample-per-participant-out tests on each of these sets. For the leave-one-sample-per-participant-out test, this meant that as more data were added to the train/test set, we adjusted the folds in the cross validation to maintain the same level of stringency. For example, on 2 days of data, the training set took 5 samples per person and tested on one sample per person, yet on 4 days of data, the training set took 11 samples per person and tested on one sample per person. The results from the leave-one-sample-per-participant-out test are presented here. We compared the median accuracy of 500 iterations of classification on real data to 500 iterations of classification on shuffled data. For the *ear*, *armpit*, and *lower back,* training and testing on 5 days yielded a permutation *P*-value of 0.002 for both uncorrected and drift-corrected data (Supplementary Fig. 4C–E; Fig. 5C–E). Using uncorrected data, a two-way ANOVA with factors of *body region* and *number of days used to train and test the model* revealed a significant effect of *body region* (*F*(2, 5988) = 25,570, *P* < 10^−5^, *ear* = 0.379 ± 0.018, *armpit* = 0.527 ± 0.022*, lower back* = 0.445 ± 0.023), and a significant effect of *number of days used to train and test the model* (*F*(3, 5988) = 7109, *P* < 10^−5^, *days 1–2* = 0.515 ± 0.027, *days 1–3* = 0.446 ± 0.02, *days 1–4* = 0.428 ± 0.018, *days 1–5* = 0.414 ± 0.014). There was also a significant interaction between the *body region* and the *number of days used to train and test the model* (*F*(6, 5988) = 1879, *P* < 10^−5^). A post-hoc Tukey’s test revealed that *lower back* performed better than *ear* (*P* < 10^−5^  *d* = 3.262), and *armpit* performed better than both (*armpit* vs *ear*: *P* < 10^−5^, *d* = 7.51, *armpit* vs *lower back*: *P* < 10^−5^, *d* = 3.696) (Supplementary Fig. 4A and B). Repeating this ANOVA with drift-corrected data revealed a significant effect of *body region* (*F*(2, 5988) = 49,280, *P* < 10^−5^, *ear* = 0.708 ± 0.014, *armpit* = 0.576 ± 0.021, *lower back* = 0.524 ± 0.021), and a significant effect of *number of days used to train and test the model* (*F*(3, 5988) = 52,097, *P* < 10^−5^, *days 1–2* = 0.756 ± 0.024, *days 1–3* = 0.612 ± 0.017, *days 1–4* = 0.545 ± 0.017, *days 1–5* = 0.497 ± 0.013). There was also a significant interaction between the *body region* and the *number of days used to train and test the model* (*F*(6, 7488) = 6910, *P* < 10^−5^). A post-hoc Tukey’s test revealed that *armpit* performed better than *lower back* (*P* < 10^−5^, *d* = 2.465), and *ear* performed better than both (*ear* vs *lower back*: *P* < 10^−5^, *d* = 10.13, *ear* vs *armpit*: *P* < 10^−5^, *d* = 7.372) (Fig. 5A and B).

To judge the impact of added days of data on overall classification accuracy, we conducted a one-way ANOVA for each *body region* on the accuracy values from 500 iterations of a leave-one-sample-per-participant-out test using each *region*’s respective classifier. When using uncorrected data, the classification accuracy initially decreased then slightly recovered for the *ear* and *armpit* as more days were added to the data set (*ear F* = 2574, *armpit F* = 755.9). With the *lower back* data, the accuracy continuously decreased as more days were added (*F* = 6869.7) (Supplementary Fig. 4A and B). A Tukey’s post-hoc test on *ear* data revealed that the accuracy decreases when training/testing the model on 3 days versus 2 days (*P* < 10^−5^, *d* = 5.338), then rises when training/testing the model on 4 days versus 3 days (*P* < 10^−5^, *d* = 0.555) and 5 days versus 4 days (*P* < 10^−5^, *d* = 0.55). Similar to the *ear*, Tukey’s test on the *armpit* showed that accuracy decreases when training/testing the model on 3 days versus 2 days (*P* < 10^−5^, *d* = 2.287), then rises for 4 days versus 3 days (*P* < 10^−5^, *d* = 0.932). However, training/testing the model on 5 days versus 4 days slightly lowers the accuracy (*P* < 10^−5^, *d* = 0.414). Contrary to the *ear* and *armpit*, Tukey’s test showed that accuracy on the *lower back* decreases consistently when training/testing the model on 3 days versus 2 days (*P* < 10^−5^, *d* = 2.116), on 4 days versus 3 days (*P* < 10^−5^, *d* = 3.85), and on 5 days versus 4 days (*P* < 10^−5^, *d* = 2.94).

When using drift-corrected data, the classification accuracy decreased for all *body regions* as more days were added to the data set (*ear F* = 6814, *armpit F* = 12,630, *lower back F* = 37,800) (Fig. 5A and B). A Tukey’s post-hoc test on *ear* data revealed that the accuracy decreases when training/testing the model on 3 days versus 2 days (*P* < 10^−5^, *d* = 4.613), on 4 days versus 3 days (*P* < 10^−5^, *d* = 2.113), and on 5 days versus 4 days (*P* < 10^−5^, *d* = 2.691). Tukey’s test on the *armpit* showed that the accuracy decreases progressively when training/testing the model on 3 days versus 2 days (*P* < 10^−5^, *d* = 6.796), 4 days versus 3 days (*P* < 10^−5^, *d* = 0.952), and 5 days versus 4 days (*P* < 10^−5^, *d* = 2.890). Tukey’s test showed that accuracy on the *lower back* also decreases when training/testing the model on 3 days versus 2 days (*P* < 10^−5^, *d* = 7.859) 4 days versus 3 days (*P* < 10^−5^, *d* = 8.139), and on 5 days versus 4 days (*P* < 10^−5^, *d* = 3.953).

### Individuals can be identified across days

**Fig. 6.**
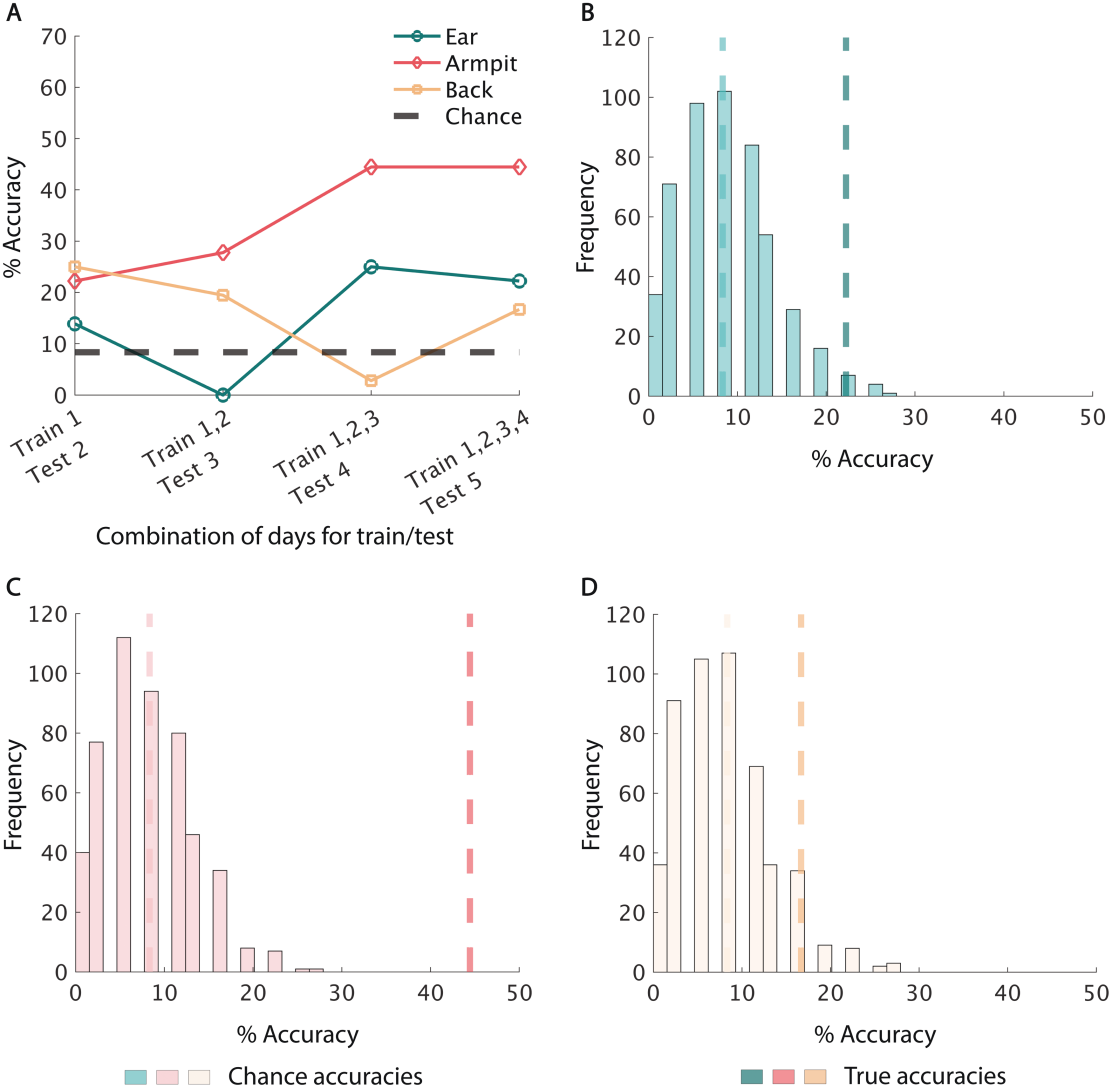
Individuals can be identified across days. A) Across-days classification accuracy of individuals using uncorrected odor from ear, armpit, and lower back trained on the first 4 days and tested on the fifth day using each body region’s best-performing respective classifier for all body regions. B–D) Distribution of accuracy values on 500 iterations of training on Days 1, 2, 3, and 4 and testing on Day 5 shuffled data with the best respective classifiers. The true accuracy value from real data is shown by the darker dashed line and the median accuracy of the shuffled data is shown by the lighter dashed line.

We sought to conduct an even stricter test where we asked: if we train a model on a group of days, can it identify a person on a new day of data? In this test, the model was trained and validated with leave-one-sample-per-participant-out cross validation. Importantly, a full day of left out data was used as an unseen test set to evaluate the performance of the trained model. As far as we know, this was not previously achieved in human identification by eNose. We took the last case, where the classifiers were trained and validated on the first 4 days of samples and tested on the unseen fifth day of samples, and compared the true accuracy value to a distribution of shuffled null data (Fig. 6B–D). Using uncorrected data, for the *ear*, training on days 1, 2, 3, and 4 and testing on day 5 yielded 22.2% accuracy (permutation *P* value = 0.012) (Fig. 6A). This same test yielded 44.4% accuracy for the *armpit* (permutation *P* value = 0.002), and 16.7% accuracy for the *lower back* (permutation *P* value = 0.046). These results show that for all *body regions*, after training on 4 days of samples, we can classify new, unseen data at above chance levels. Unlike in the within-day tests, in the across-days comparison, correcting for drift using the control odor in fact lowered rather than improved performance (Supplementary Fig. 5).

## Discussion

In this study, we sought to determine whether we could use standard eNose technology to identify people by the smell of their ear. We also tested two other body regions, armpit, and lower back, for their efficacy in personal identification from odor. We found that sampling any of the 3 body regions can distinguish between 12 individuals significantly above chance. When we learned and tested on data from the same day, performance was remarkably good for all body regions. Ear was indeed slightly better than armpit and back when we used our a-priori selected classifier (Fig. 3A and B), but this advantage was negated when we optimized classifiers for each body region, resulting in lower back outperforming ear and armpit (Fig. 3C and D). In turn, the ear advantage reemerged as more data was added. In other words, if we combine data across days to form a virtual day of extensive testing, the ear stands out (Fig. 5A and B). This provides some support for our hypothesis of higher stability in the ear-based odor source—increasing data set size reduced noise in ear more than in armpit and back. However, in contrast to the biometric-quality within-day data, performance across days was relatively poor. After 4 days of training on *ear* data, testing on a separate fifth and new day of data pushed performance down from ~87% to ~22% accuracy, a result still well above chance, but not biometric. In turn, armpit outperformed ear and lower back on the cross-day novel data test, achieving 44.4% accuracy. To nevertheless place these underwhelming results in a more positive light, previous efforts to identify humans by smell with an eNose could only identify when using all the available data (Wongchoosuk et al. 2009, 2011; Zheng et al. 2019). As far as we know, identification of newly acquired data (as in Fig. 6) was not previously achieved in an effort to identify humans by smell.

Although significant, this level of performance remains insufficient for a biometric tool. Why did cross-day performance deteriorate from within-day performance to this extent? Our study was conducted in a naturalistic setting. We did not collect samples from the body and submit them to testing in a controlled environment (e.g. temperature- and humidity-controlled testing vials), but rather conducted real-time sampling in 50 s or less. As a result, the overall extent of odor, and hence eNose signal, was very low in this experiment. Moreover, the experiment was conducted in a typical room fluctuating temperature and humidity conditions that significantly impact eNose readings. Finally, even under the most controlled (non-naturalistic) conditions, there is still drift in both actual body odor and in eNose sensor performance (Supplementary Fig. 1). We made efforts to remove this drift from the signals by dividing all sample endpoints by their matched fresh control sample endpoints. This yielded a significant increase in the accumulated days’ classification accuracies, improving the last case of training and testing on all 5 days for the ear from 37.9% to 70.8% on a leave-one-sample-per-participant-out test. Similar increases in accuracies were also seen for the other body regions. Nevertheless, performance remained relatively poor across days (we note that we stumbled across a drift-correction method that provided for better cross-day results; specifically, dividing all data by the ear day-1 baseline. However, because we have no rationale to justify this approach, we present it as an anecdote in Supplementary Fig. 6, hinting at potential levels of improvement in performance that may be attainable with a better understanding of these signals). This level of performance may be improved in the future by better computational tools (Kermani et al. 1999; Lötsch et al. 2019) (we reiterate that the entire raw data set is available for download such that members of the community may test alternative approaches), and by better eNose hardware optimized for the very low level of VOCs involved in body odor sampling (Jha and Yadava 2011; Sabri and Alfred 2018). Taken together, we conclude that what we provide here is an affirmative proof of concept: humans can be identified by the smell in their ear, and that sampling ear odor may provide some practical advantages over other body parts.

## Supplementary Material

bjad053_suppl_Supplementary_Material

bjad053_suppl_Supplementary_Data

## Data Availability

All data are available for download in Data File 1.
