# Supplementary material for: An electronic nose can identify humans by the smell of their ear
Source: Chem Senses. 2024 Jan 18;49:bjad053. doi: 10.1093/chemse/bjad053 (PMC10810274; doi:10.1093/chemse/bjad053)
Supplement: bjad053_suppl_Supplementary_Material [file bjad053_suppl_supplementary_material.docx]

Supplementary Materials for:

**An Electronic Nose Can Identify Humans by The Smell of Their Ear**

Stephanie Brener*, Kobi Snitz, Noam Sobel

The Azrieli National Center for Human Brain Imaging and Research and the Department for Brain Sciences, Weizmann Institute of Science, Rehovot, Israel.

**Data File 1**

Data File 1 contains all the raw data given as a MATLAB structure. Each row corresponds to a participant’s sample of either baseline, control, ear, armpit, or lower back odor. The data are in alphabetical order corresponding to the participant’s codename and then the sample name (e.g. ear, armpit, etc.). The order in which the participants were measured each day is described by Supplementary Table 1. The order in which body regions were sampled each day can be seen in Supplementary Table 2. The first 900 rows correspond to the data from eNose 1 and the second set of 900 rows correspond to the data from eNose 2 in the exact same order. Analysis was done by first concatenating together the endpoints from both eNoses. Each row in the structure contains the following fields:

- filename: the name of the sample, including which participant and body region
- info: the sample information provided by the eNose’s built-in program
- data: the raw data matrix of 50x10 (51x10 in some cases) timepoints by sensors
- EndptsVector: the vector of sensor endpoints used in all training and testing
- TimeOfDay: the order in which that person was sampled each day

The naming convention of the filenames corresponds to the following:

- SubjectCode_Date_BodyRegion_#OfSample.nos

For example: This filename, 'M758_20220313_Armpit_R_1.nos', indicates the following*:

- Subject code: M758
- Date taken: March 13, 2022
- Body region: Right Armpit
- # of sample: 1

The possible sample types and their meanings are as follows:

1. Armpit_R: Right armpit
2. ArtificialCerumen: Overnight control odor
3. ArtificialCerumen_Fresh: Fresh control odor
4. Back: Lower back
5. Baseline: Baseline room odor
6. Ear_R: Right ear

| **Time of Day** | **Sunday** | **Monday** | **Tuesday** | **Wednesday** | **Thursday** |
| --- | --- | --- | --- | --- | --- |
| **8am** | M760 | M760 | W907 | M760 | M760 |
| **9am** | W909 | M758 | W905 | M758 | M758 |
| **10am** | M758 | W905 | M758 | eNose off | W909 |
| **11am** | W906 | W906 | M760 | eNose off | W906 |
| **12pm** | W905 | W878 | W909 | eNose off | W908 |
| **1pm** | W908 | M761 | W908 | W906 | W878 |
| **2pm** | M761 | W909 | W878 | W909 | W904 |
| **3pm** | W907 | W907 | W906 | W907 | W907 |
| **4pm** | W904 | W908 | M761 | W908 | W910 |
| **5pm** | W910 | M759 | W904 | W910 | M759 |
| **6pm** | W878 | W904 | W910 | M759 | M761 |
| **7pm** | M759 | W910 | M759 | W904 | W905 |
| **8pm** |  |  |  | W905 |  |
| **9pm** |  |  |  | W878 |  |
| **10pm** |  |  |  | M761 |  |

**Supplementary** **Table 1:** Sampling times by time and day. Each code is a unique participant; M codes are men and W codes are women.

| **Day of the week** | **Order of sampling** |
| --- | --- |
| Sunday | Ear, Armpit, Back |
| Monday | Ear, Back, Armpit |
| Tuesday | Armpit, Ear, Back |
| Wednesday | Armpit, Back, Ear |
| Thursday | Back, Ear, Armpit |

**Supplementary** **Table 2:** Order of body-region sampling by day.


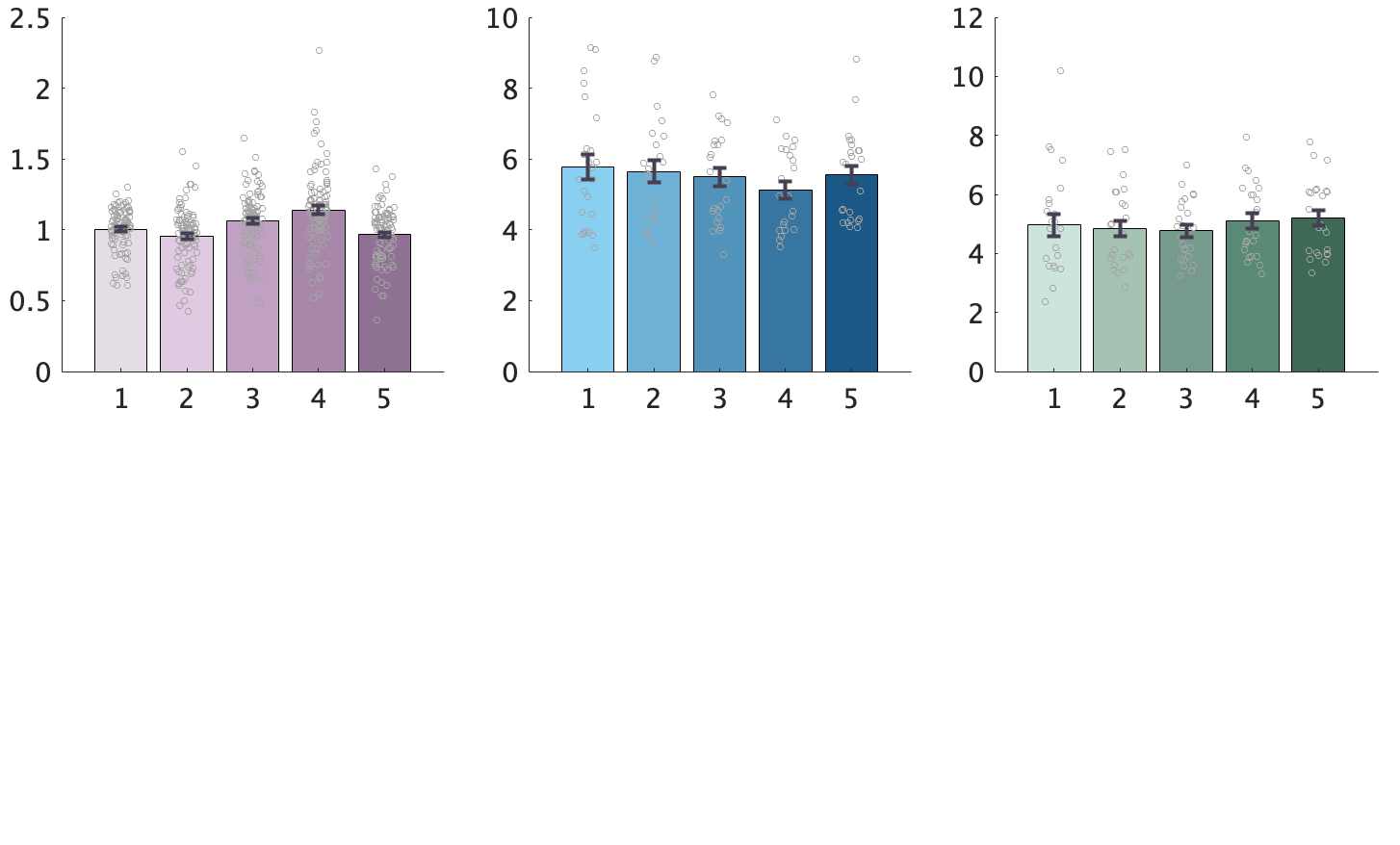

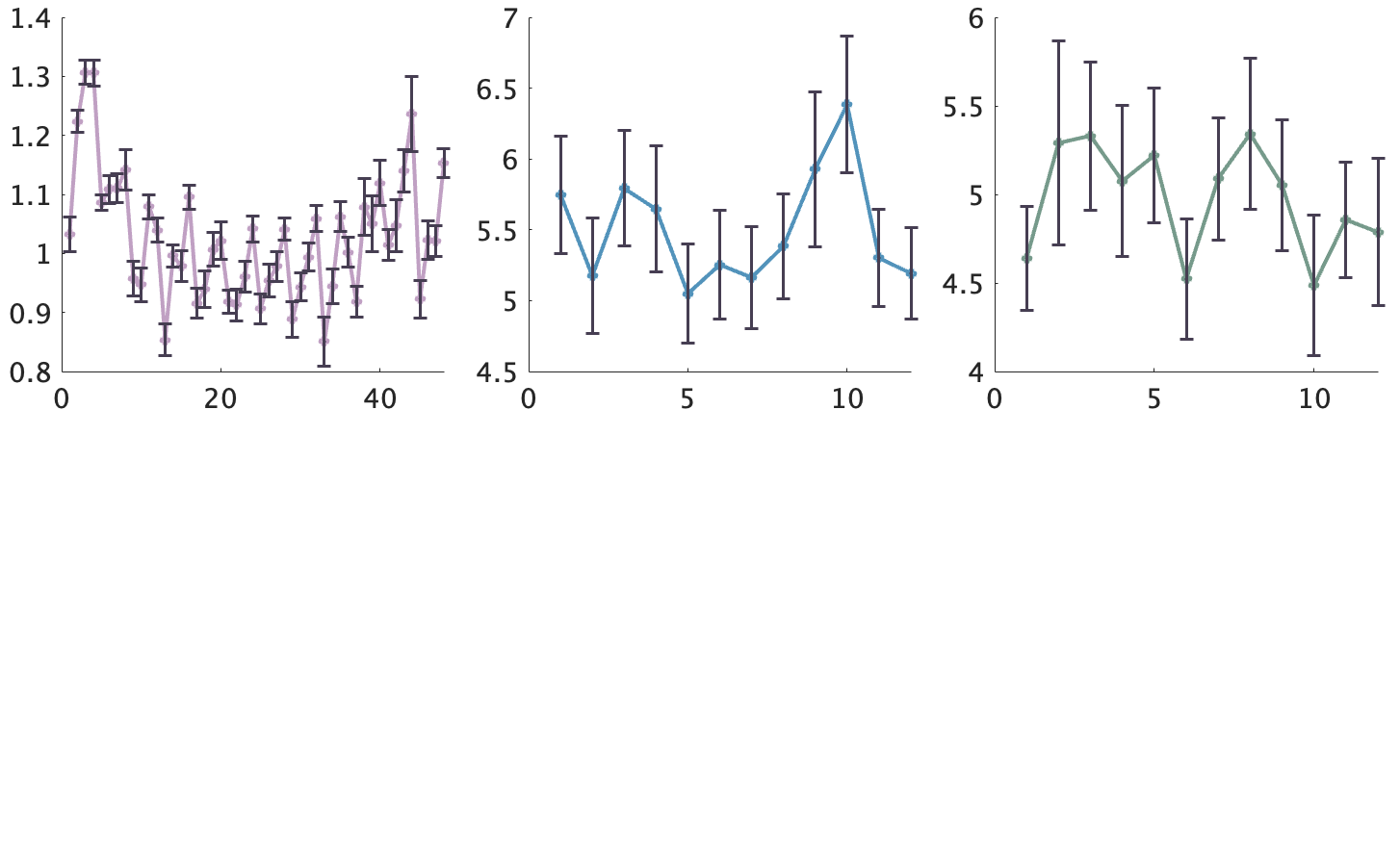


Sensor 7 change in conductivity (G/G0)

A

B

C

D

E

F

Day

Sample # during single day

p = 0.004

p < 0.001

p = 0.019

p = 0.004

p < 0.001

p = 0.045

**Supplementary Figure 1: eNose sensors undergo across days and within day drift**

A. Bar plot showing the average endpoint value of sensor 7 averaged across all 48 baseline odor samples from both eNoses for each day. The 96 values (48 from each eNose for every hour of the day) are plotted over the average, and standard error bars are shown. Sensor 7 was chosen for visualization because it was the most sensitive sensor with maximal activity during measurement. A repeated measures ANOVA (rmANOVA) with the Huynh-Feldt sphericity correction found a significant effect of day in the baseline samples (F(1.3, 24.5) = 7.808, p = 0.006), with four significant pair-wise interactions shown on the plot. B. Bar plot of the average sensor 7 endpoint value averaged across 24 overnight control odor samples (12 from each eNose) in each day. The rmANOVA revealed a significant effect of day in the overnight control odor samples (F(1.38, 26.21) = 5.073, p = 0.023), with two significant pair-wise interactions between days shown on the plot. C. Bar plot of the average sensor 7 endpoint value averaged across 24 fresh control odor samples in each day. The results of the rmANOVA showed no significant effect of day for the fresh control odor samples (F(2.316, 44.0) = 1.008, p = 0.383). The results from A-C indicate drift across days, which may be due to day 4 having slightly different measurement conditions since it was after a night of rain (shifting ambient humidity levels) and the eNose was off for three hours during the day. D. Plot of the ordered 48 baseline odor samples from sensor 7 taken during the day averaged over the five days and both eNoses with standard error bars. An rmANOVA indicated a significant effect of hour of the day for the baseline odor samples (day 1 (F(1.469, 27.91) = 3.792, p = 0.047), day 2 (F(1.474, 28.01) = 5.172, p = 0.019), day 3 (F(1.311, 24.91) = 12.42, p < 0.001), day 4 (F(1.091, 20.73) = 6.971, p = 0.014), and day 5 (F(1.344, 25.54) = 5.136, p = 0.023)). E. Plot of the 12 overnight control odor samples from sensor 7 in order from morning to night averaged over five days and both eNoses. The rmANOVA showed a significant effect of hour of the day for the overnight control odor samples (day 1 (F(1.285, 24.42)=7.5, p=0.007), day 2 (F(1.068, 20.30)=8.683, p=0.007), day 3 (F(1.513, 28.75) = 6.781, p < 0.008), day 4 (F(1.389, 26.40) = 4.911, p = 0.025), and day 5 (F(1.057, 20.08) = 6.123, p = 0.021)). F. Plot of the 12 fresh control odor samples from sensor 7 ordered and averaged over five days and both eNoses. The rmANOVA yielded a significant effect of hour of the day for the fresh control odor samples as well (day 1 (F(1.375, 26.12) = 4.339, p < 0.036), day 2 (F(1.14, 21.66) = 5.242, p = 0.028), day 3 (F(1.729, 32.85) = 6.009, p < 0.008), day 4 (F(1.136, 21.59) = 5.783, p < 0.022), and day 5 (F(1.094, 20.79) = 6.176, p = 0.019)). The results of D-E indicate within day sensor drift. However, for the fresh control odor, most of the significant pairwise differences between any two hours of the day occurred between the first few hours of sampling and the last few hours of sampling. This may be indicative of the eNoses’ internal state after a long period of inactivity and a long period of use.


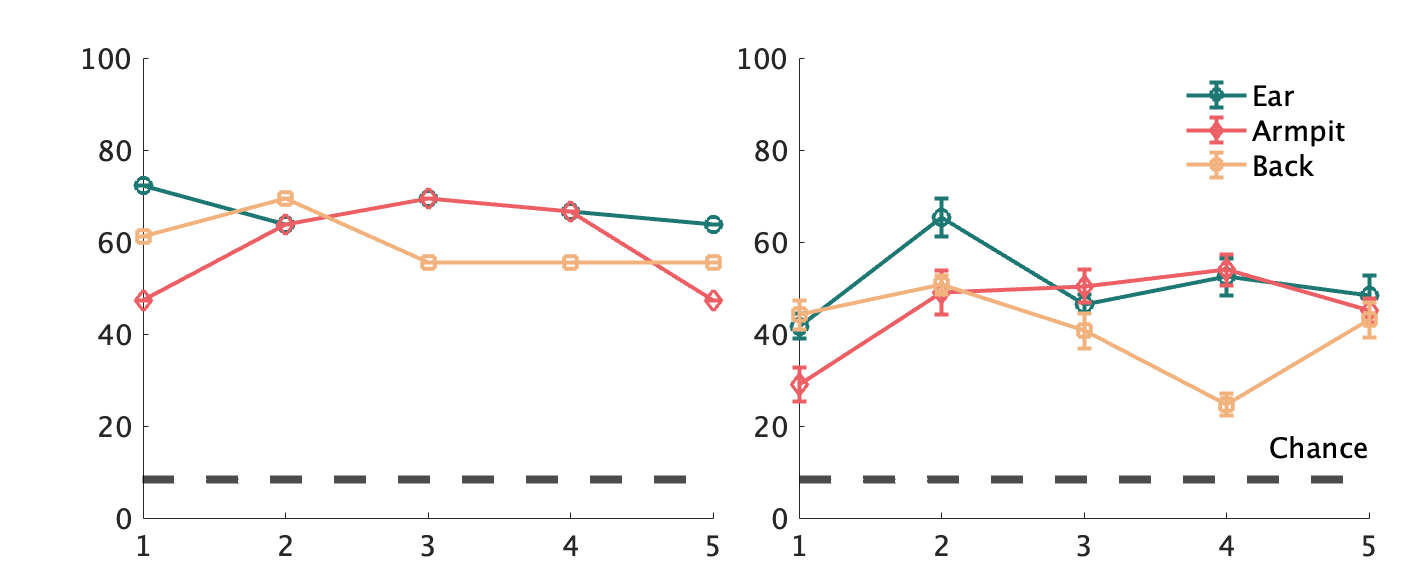

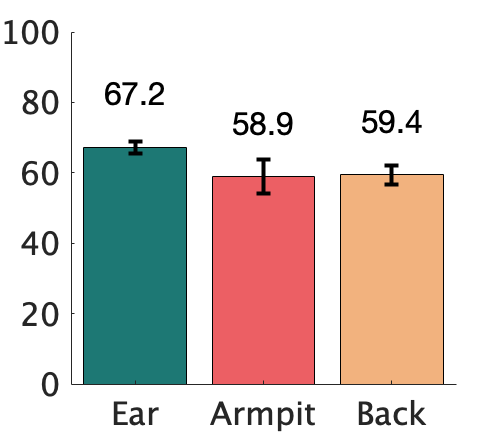

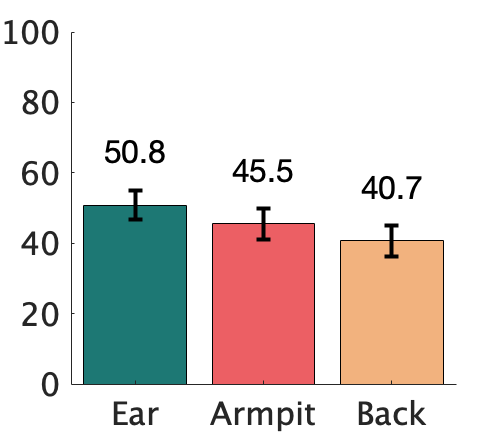


Day

Day

% Accuracy

A

B

**Supplementary Figure 2: Individuals can be identified across samples within a day before optimizing for drift**

**A.** Mean classification accuracy with standard deviation of individuals using the odor from ear, armpit, and lower back when trained and tested within single days using the Fine KNN classifier in a leave-one-out test for all body regions with no drift correction. The overall average accuracy with standard error bars for ear, armpit, and back is shown as an insert in each panel. The chance level prediction accuracy is given by the black dashed line at 8.3%. **B.** Within-day mean classification accuracy under the same conditions but for the 3-fold cross validation case plotted with standard deviation.


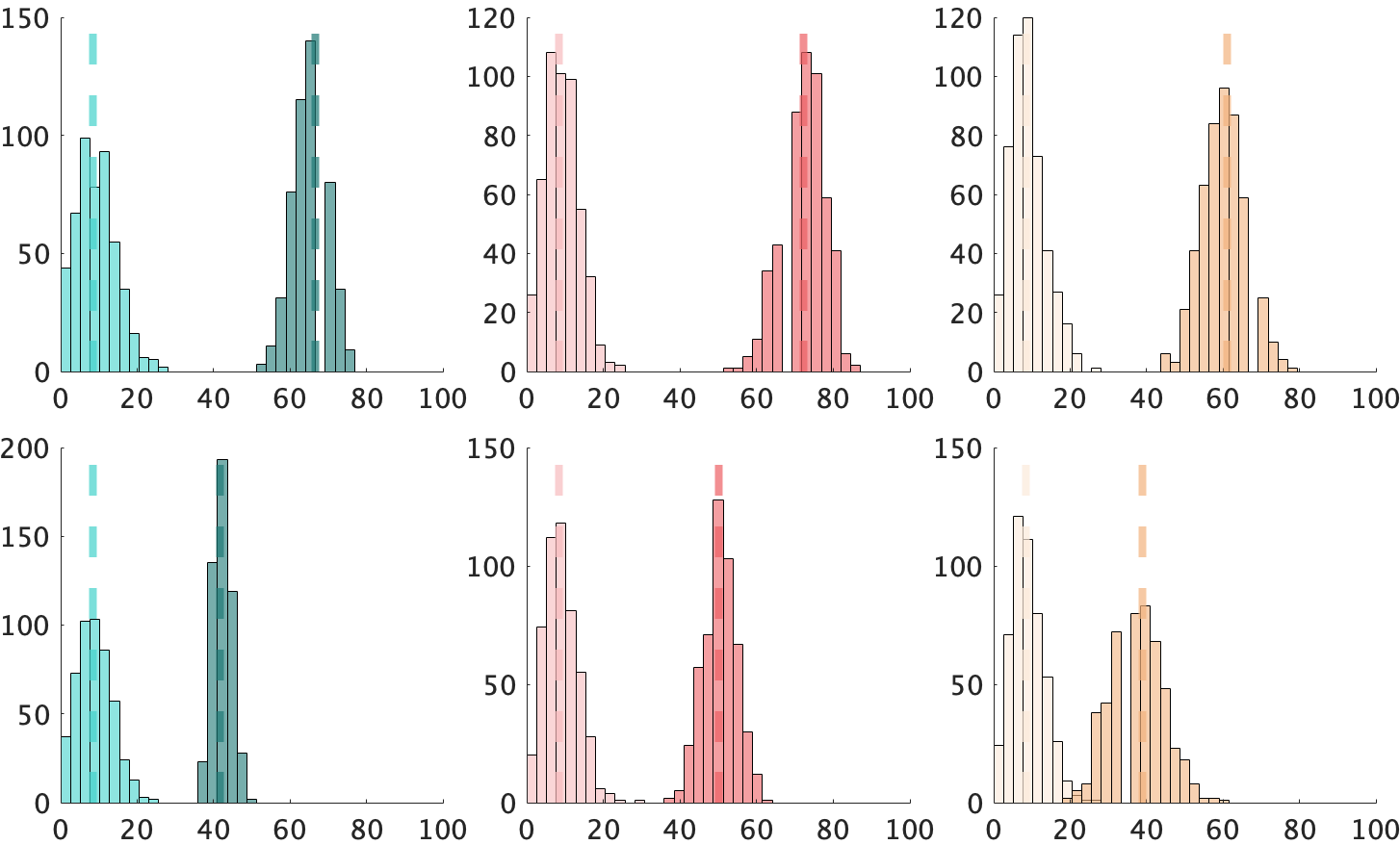

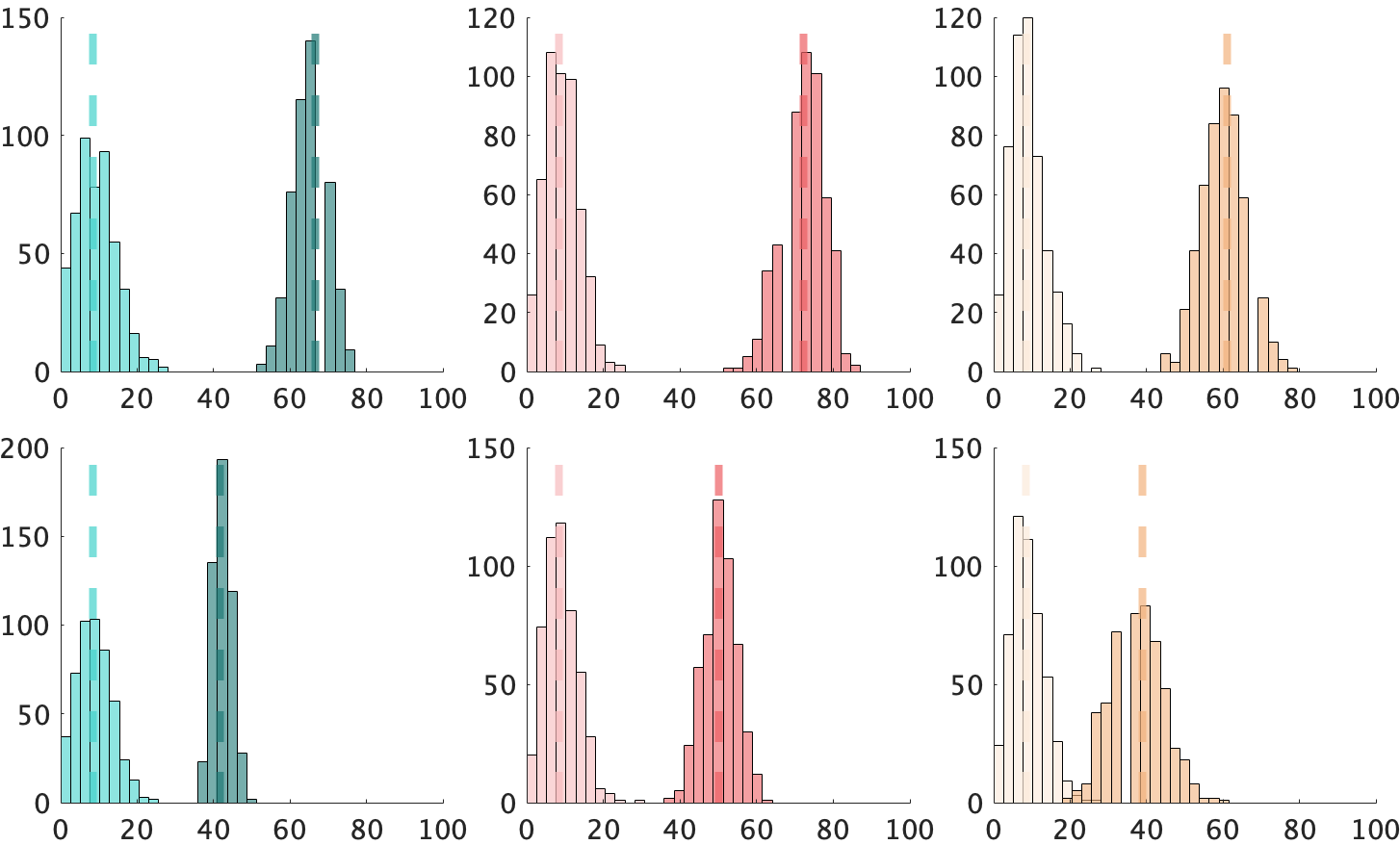


Frequency

A

B

C

D

E

F

% Accuracy

Chance accuracies

True accuracies

**Supplementary Figure 3: Within single day performance was significantly better than chance with best classifiers and no drift correction**

**A-C.** Distributions of accuracy values on 500 iterations of training/testing with the Fine KNN classifier on non-corrected real data on the right and shuffled data on the left for ear, armpit, and lower back respectively from the best-performing day (blue is *ear*, red is *armpit*, and yellow is *lower back*). Median accuracy values are displayed in dashed lines. **D-F.** Distributions of accuracy values under the same conditions but for each body region’s worst-performing day.


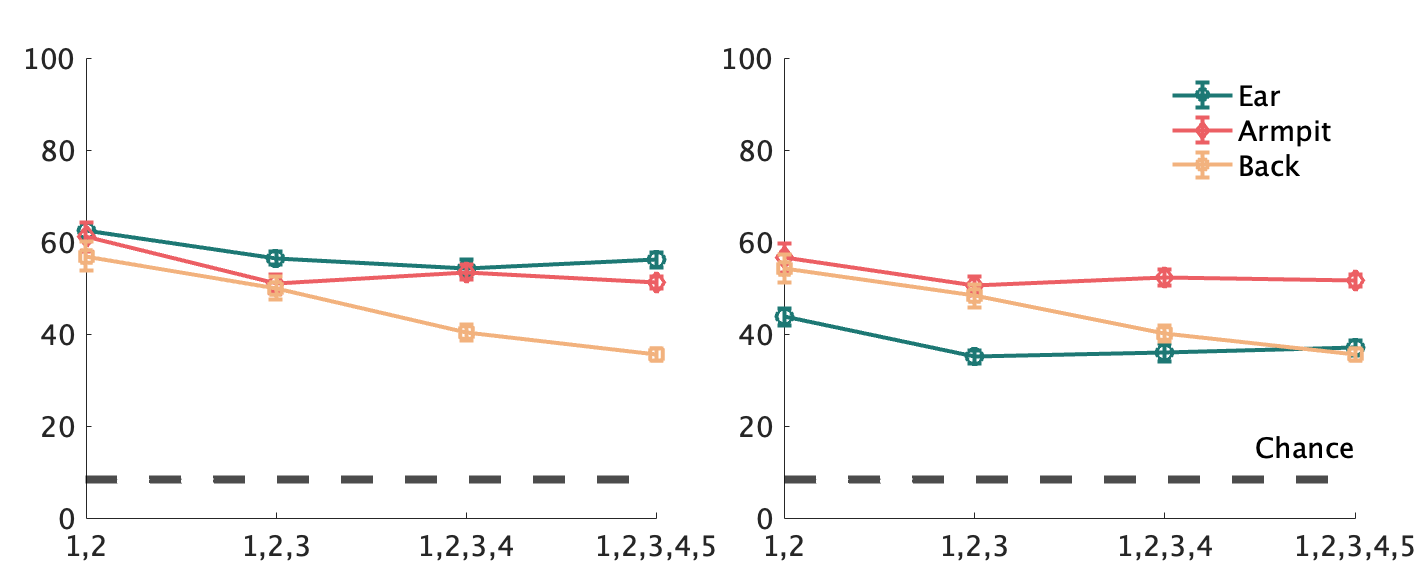

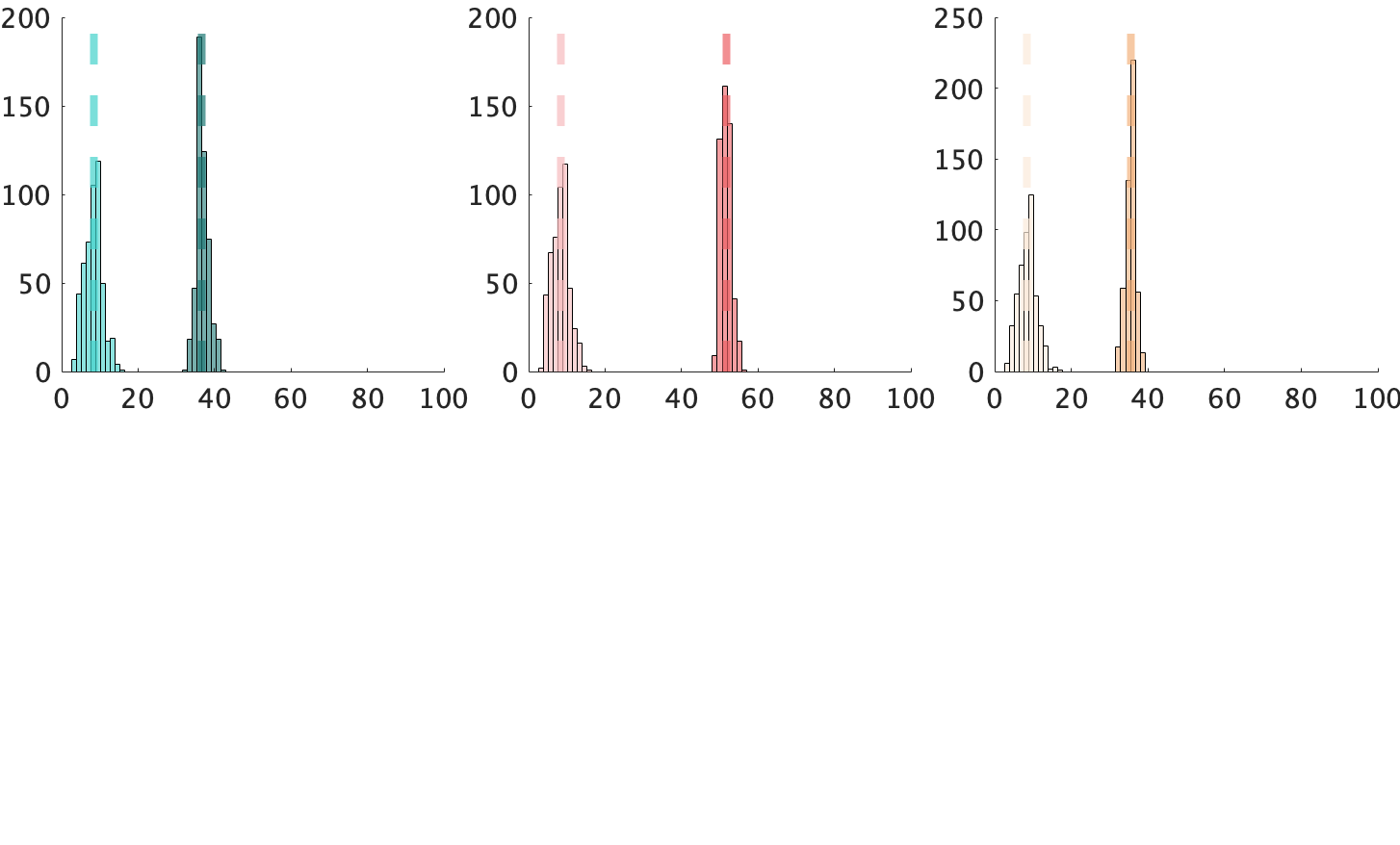


A

B

% Accuracy

Days used for train and test

Days used for train and test

Frequency

C

D

E

% Accuracy

Chance accuracies

True accuracies

**Supplementary Figure 4: There is an ear advantage as data accumulates even without drift correction**

**A.** Mean across accumulated days classification accuracy of individuals using the odor not corrected for drift from ear, armpit, and lower back trained and tested on multiple days with each body region’s best performing respective classifier in a leave-one-out test for all body regions. **B.** Mean across accumulated days classification accuracy under the same conditions but for the stricter leave-one-sample-per-participant-out cross validation test, with standard deviation. **C-E.** Distributions of accuracy values on 500 iterations of training/testing with a leave-one-value-per-participant-out test on accumulated Days 1, 2, 3, 4, and 5 with the best respective classifiers on real data on the right and shuffled data on the left for ear, armpit, and lower back respectively. Median accuracy values are displayed in dashed lines.


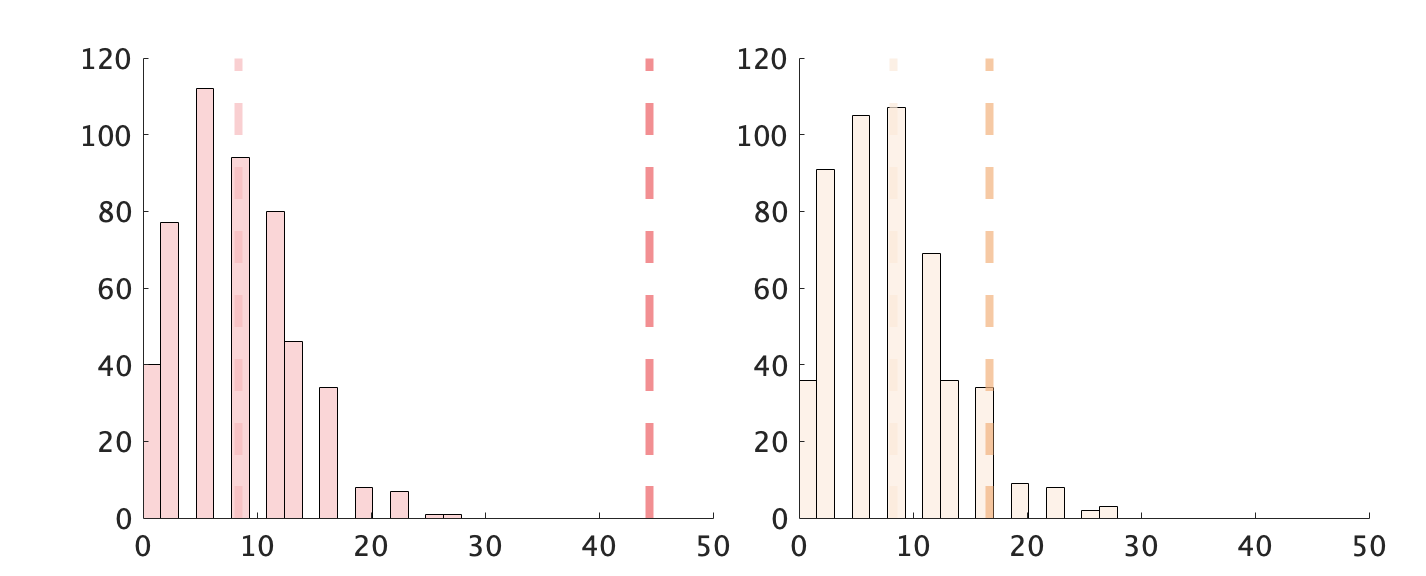

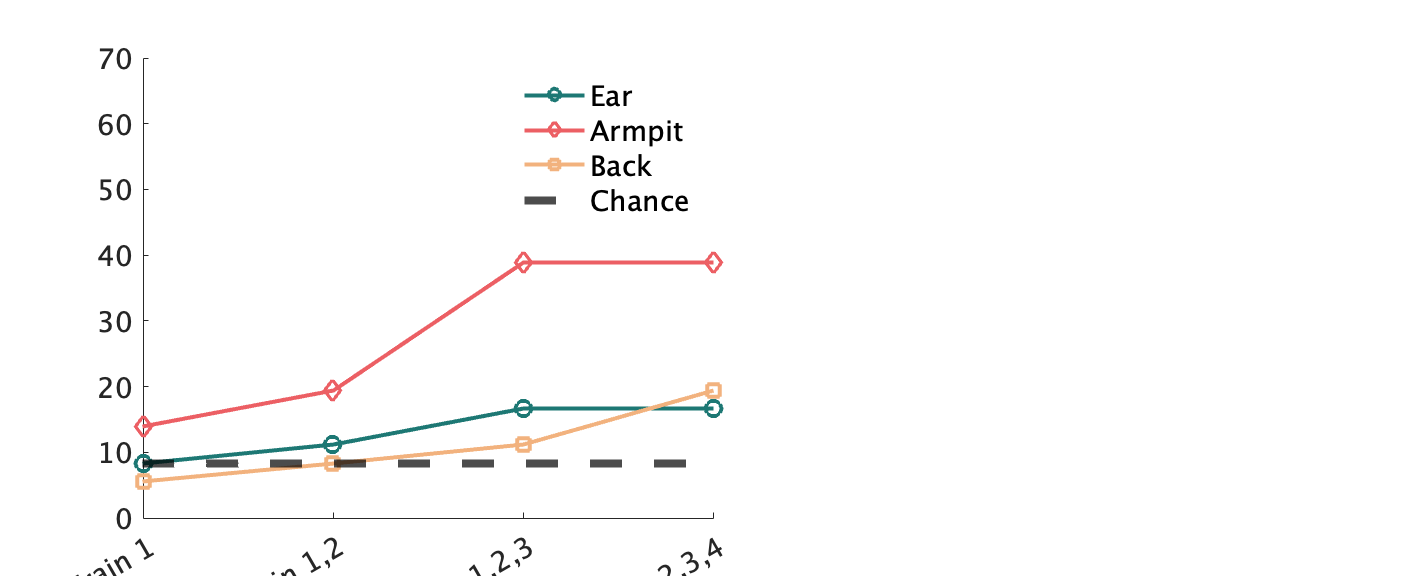

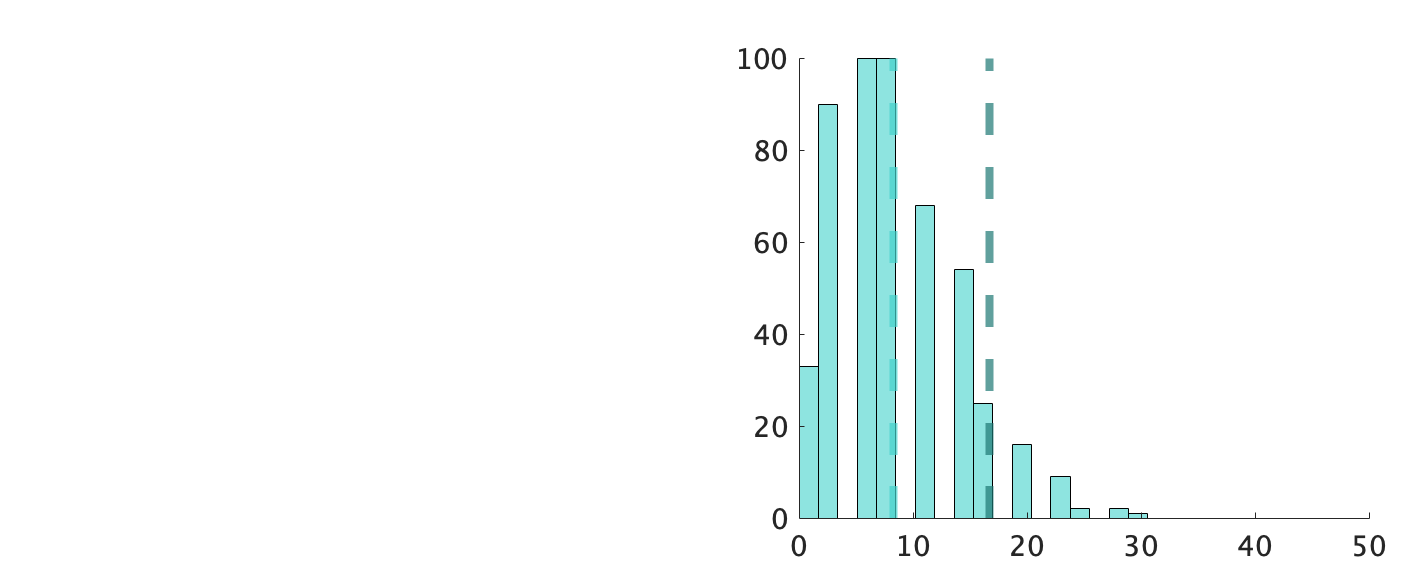

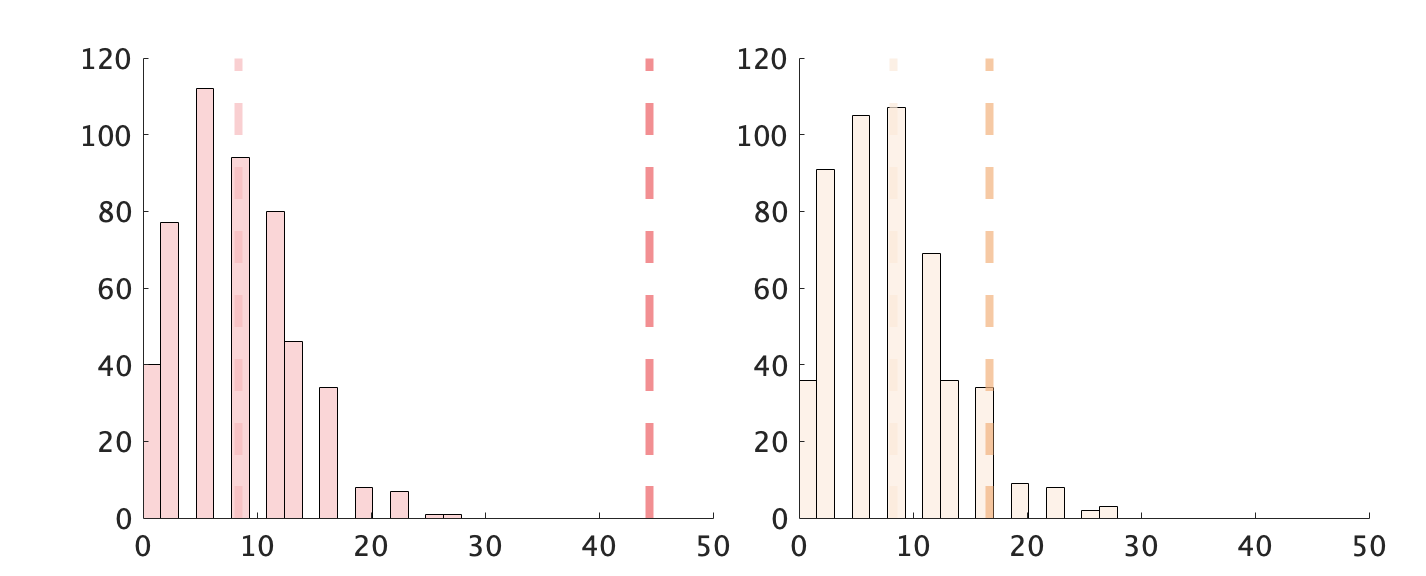

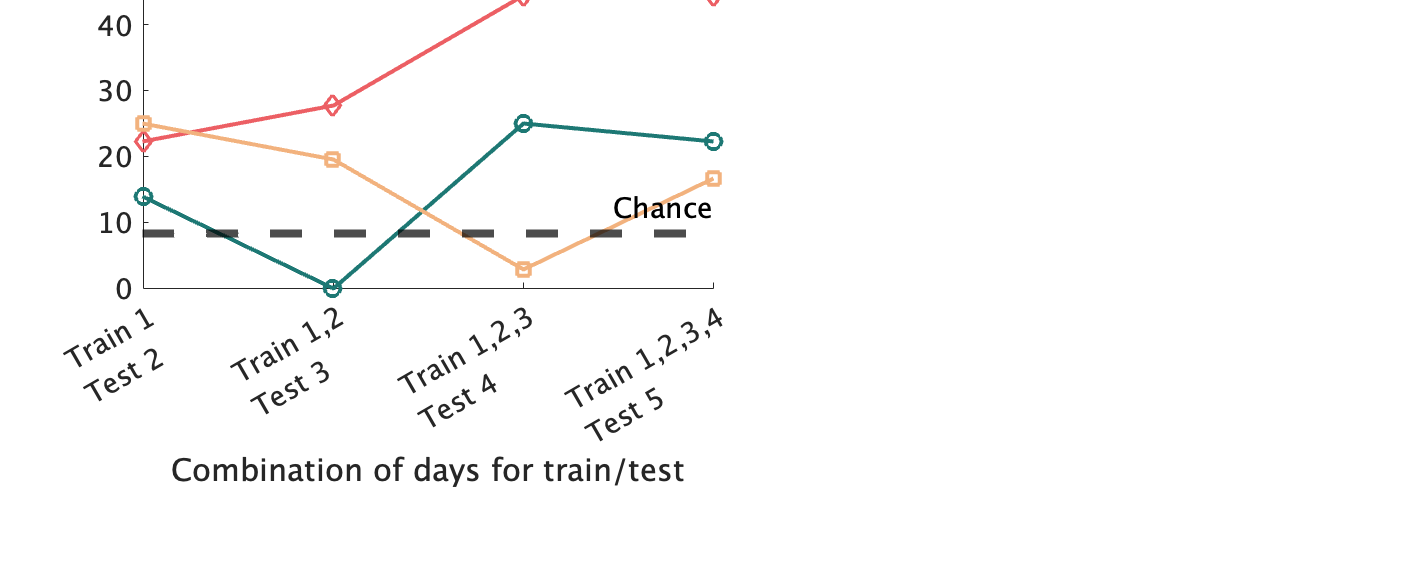


Combination of days for train/test

A

B

C

D

% Accuracy

Frequency

Frequency

Frequency

% Accuracy

% Accuracy

Accuracy

Chance accuracies

True accuracies

**Supplementary Figure 5: Individuals can be identified across days**

A. Across-days classification accuracy of individuals using corrected odor from ear, armpit, and lower back trained on the first four days and tested on the fifth day using each body region’s best performing respective classifier for all body regions. B-D. Distribution of accuracy values on 500 iterations of training on Days 1, 2, 3, and 4 and testing on Day 5 shuffled data with the best respective classifiers. The true accuracy value from real data is shown by the darker dashed line and the median accuracy of the shuffled data is shown by the lighter dashed line. *Ear* performed with 16.7% accuracy (p = 0.0619), *armpit* with 38.9% accuracy (p = 0.002), and *lower back* with 19.4% accuracy (p = 0.028).


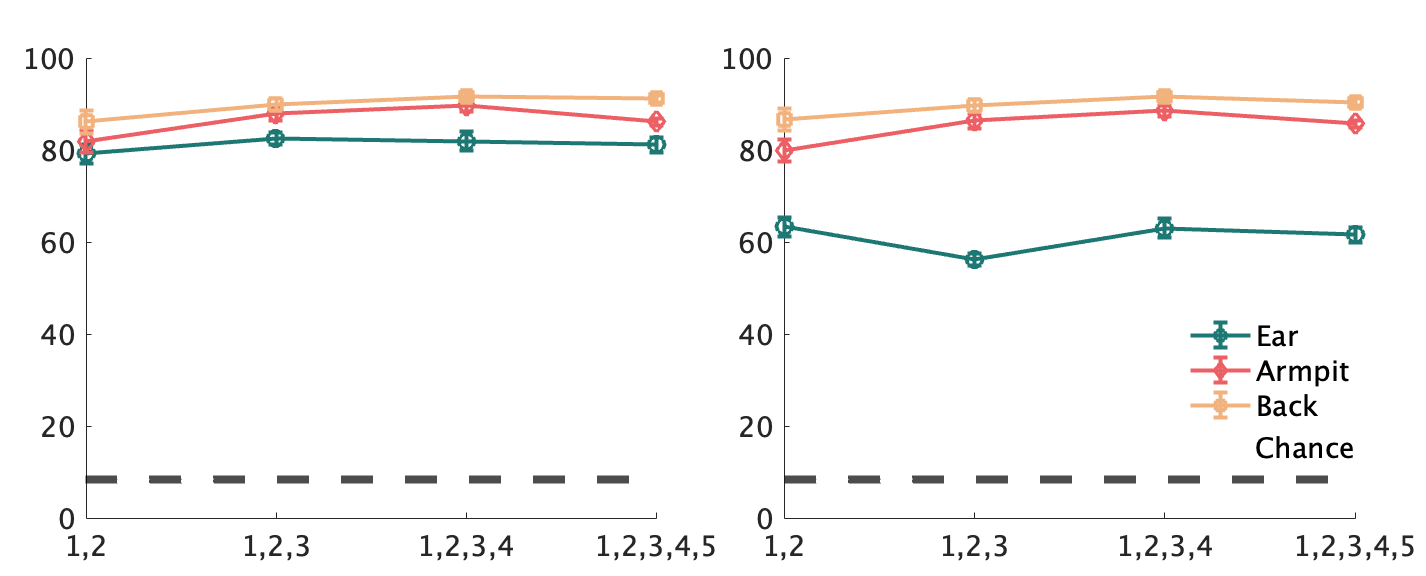

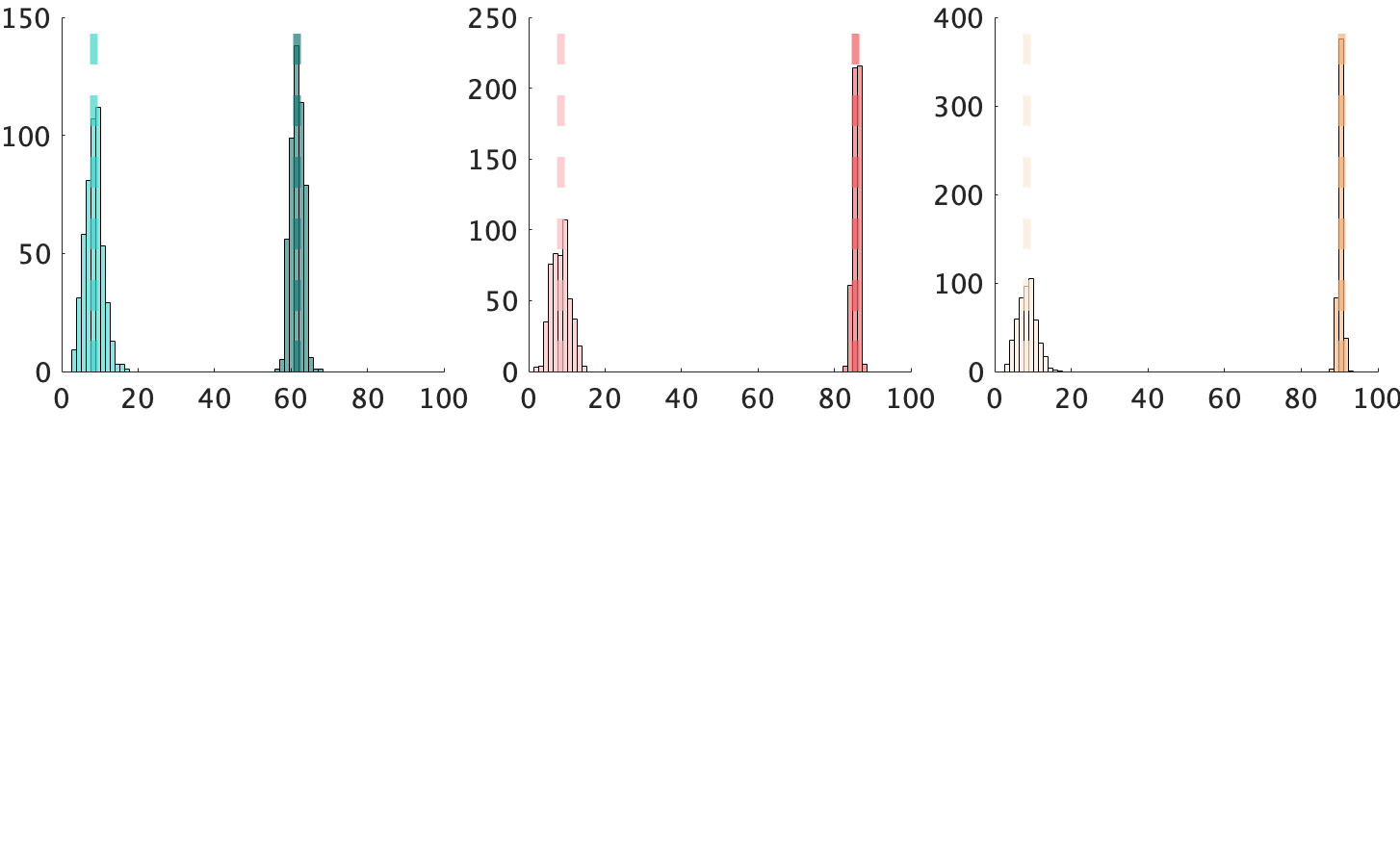


A

B

% Accuracy

Days used for train and test

Days used for train and test

Frequency

C

D

E

% Accuracy

Chance accuracies

True accuracies

**Supplementary Figure 6: Accuracy improves for all body regions after drift-correcting with the ear day 1 baseline odor**

**A.** Mean across accumulated days classification accuracy of individuals after correcting for drift by dividing ear, armpit, and lower back odor by their matched ear day 1 baseline samples. Data was trained and tested on multiple days with each body region’s best performing respective classifier in a leave-one-out test for all body regions. **B.** Mean across accumulated days classification accuracy under the same conditions but for the stricter leave-one-sample-per-participant-out cross validation test, with standard deviation. **C-E.** Distributions of accuracy values on 500 iterations of training/testing with a leave-one-value-per-participant-out test on Days 1, 2, 3, 4, and 5 with the best respective classifiers on real data on the right and shuffled data on the left for ear, armpit, and lower back respectively. Data was drift-corrected by dividing all samples by their matched ear day 1 baseline sample. Median accuracy values are displayed in dashed lines.
